# Angiotensin II induces sex-specific ventricular remodeling in aging C57Bl/6 mice

**DOI:** 10.1007/s11357-025-01924-y

**Published:** 2025-11-07

**Authors:** Ninh Khuong, Manish Mishra, Shubham Banga, Susan E. Howlett

**Affiliations:** 1 Department of Pharmacology, Dalhousie University, Halifax, NS Canada; 2 Department of Medicine (Geriatric Medicine), Dalhousie University, PO Box 15000, Halifax, NS B3H 4R2 Canada

**Keywords:** Hypertension, Hypertrophy, Sex differences, Inflammation, Heart failure

## Abstract

Renin-angiotensin system (RAS) activation in heart failure (HF) increases circulating angiotensin II (AII). This increases blood pressure (BP) and promotes adverse ventricular remodeling that makes HF worse. Although human HF increases with age and differs between the sexes, preclinical studies have used young, mostly male animals. We investigated sex differences in AII-induced cardiac remodeling in aging C57BL/6 mice. Mice (≈16 months) were infused with AII (3 mg/kg/day; 6 weeks; osmotic minipumps). BP (tail cuff), ventricular structure/function (echocardiography), M1 (Cd80/Mcp-1/Cd68), and M2 (Mrc1) macrophage markers and profibrotic markers (transforming growth factor beta (Tgfb1), collagen 1 (Col1a1), collagen 3 (Col3a1); qPCR/Western blot) were measured. AII treatment increased BP and heart weight/tibia length in both sexes. Interestingly, AII increased wall thickness in females but reduced it and caused left ventricular (LV) dilation in males only. AII increased E/A ratios in males and isovolumic relaxation time in both sexes, indicative of diastolic dysfunction. However, isovolumic contraction time (IVCT) increased (12.0 ± 0.7 vs. 24.3 ± 3.0 ms; *p* = 0.0001) and ejection fraction (EF) declined in males (68.9 ± 1.9 vs. 46.1 ± 4.5; *p* = 0.0001), which indicates marked systolic dysfunction. By contrast, IVCT was unaffected, and EF declined but remained well above 50% in females (68.1 ± 1.3 vs. 55.9 ± 2.0; *p* = 0.01). mRNA for macrophage markers (Cd80, Mrc1), Tgfb1, Col1a1, and Col3a1, plus collagen 1 protein increased in treated male ventricles. By contrast, AII increased collagen 3 protein in females compared to treated males. Thus, AII promotes inflammatory/profibrotic signaling, which contributes to fibrosis and impairs systolic function in aging males. Older females appear resistant to these effects.

**Graphical Abstract:**

**Figure:**
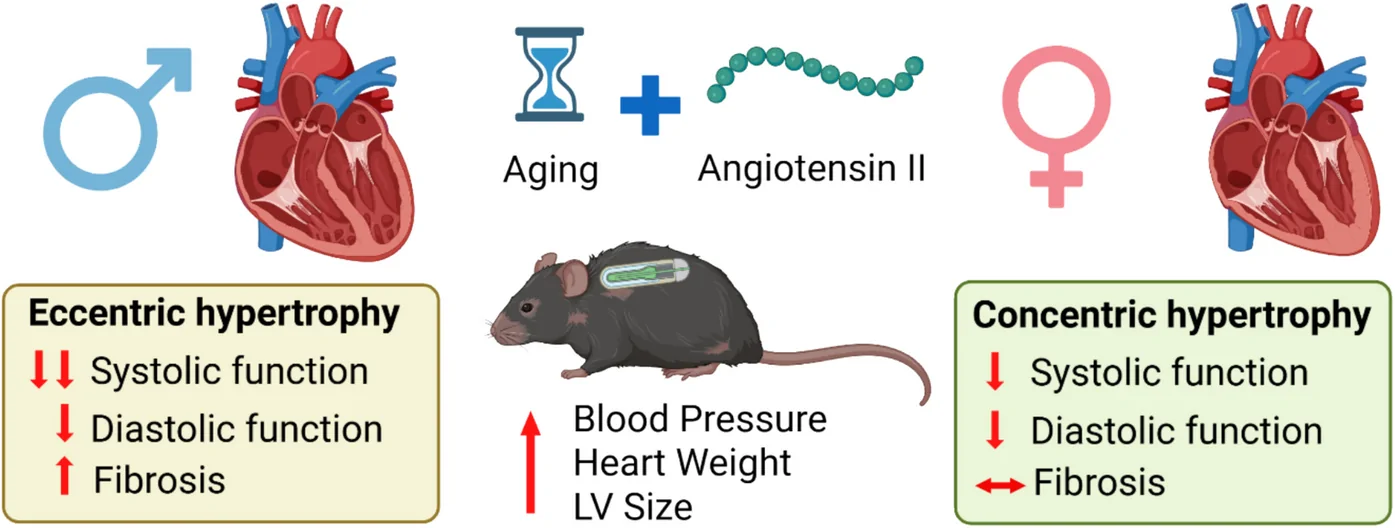

**Supplementary Information:**

The online version contains supplementary material available at https://doi.org/10.1007/s11357-025-01924-y.

## Introduction

Heart failure (HF) is a clinical condition characterized by a decrease in the ability of the heart to pump blood [1, 2]. This decrease in contractile function leads to chronic activation of the renin-angiotensin system (RAS) both in humans and in animal models of this condition [3]. This, in turn, increases circulating levels of angiotensin II (AII), which elevates blood pressure (BP) [3]. High BP promotes inflammation [4] and enhances fibrotic signaling [5] via pro-inflammatory cytokines and profibrotic mediators at the organ and tissue levels [6–9]. This contributes to adverse remodeling of the failing heart [3]. Indeed, experimental studies demonstrate that treatment with drugs that block the RAS slows the progression of damage in vital organs, including the heart [10–12]. Therefore, drugs that inhibit the RAS, including AII receptor antagonists and angiotensin-converting enzyme inhibitors, are commonly used to treat HF clinically [13]. Despite advances in treatment, however, HF remains an important cause of cardiovascular morbidity and mortality globally [1, 2].

Advanced age is a major risk factor for the development of HF [14, 15]. Indeed, the aging process leads to cellular, structural, and functional changes in the heart, and these changes may make older adults more susceptible to the development of diseases like HF [16–18]. For example, both systolic and diastolic dysfunction are prominent in aging hearts, and at the cellular level, even in the absence of overt cardiovascular disease [19, 20]. There is also evidence that the RAS is activated in aging rodents, and that receptors for AII are upregulated in the ventricles [21]. In support of this, RAS inhibition can improve cardiac structure and function in both young and aging mouse models [22, 23]. In addition, ablation of the gene for the AII type 1 receptor protects the mouse heart from fibrosis and hypertrophy [24]. Thus, age-associated adverse remodeling in the heart, in association with activation of the RAS, may facilitate the development of HF in older adults.

There is emerging evidence that age-related changes in the cardiovascular system, a phenomenon known as cardiac aging, can differ between the sexes [17, 25, 26]. Sex-specific changes associated with cardiac aging may promote the development of different cardiac diseases in older males and females [17, 25, 26]. For example, there is evidence that aging promotes the loss of ventricular myocytes in males but not females, and this can disrupt cardiac contractile function [17]. Indeed, male-specific loss of myocytes is thought to contribute to the development of systolic dysfunction and a condition known as HF with reduced ejection fraction (EF) or HFrEF [17]. In support of this, the prevalence of HFrEF is higher in men than in women, at least until 80 years of age [27]. Aging also is associated with thicker left ventricular walls, especially in older females [17]. This may help explain why HF with preserved EF (HFpEF; EF > 50%) is more common in older women than in men [28].

Despite nascent evidence for male–female differences in cardiac aging and cardiovascular disease expression, mechanistic studies of adverse cardiac remodelling in response to AII have investigated effects in young, mostly male animals. Here we investigated whether adverse ventricular remodeling induced by chronic exposure to pressor doses of AII differed between the sexes in older C57BL/6 mice. The impact of chronic AII infusion on cardiac structure and function in vivo was assessed with echocardiography. Impacts of chronic AII infusion on M1 (Cd80, Mcp-1, Cd68) and M2 (Mrc1) macrophage markers and profibrotic markers (transforming growth factor beta, collagen 1, collagen 3) in the ventricle were assessed with quantitative polymerase chain reaction (qPCR) and/or Western blotting.

## Methods

### Animals

Male and female C57BL/6 mice were purchased from Charles River Laboratories (St. Constant, QC) and were aged in the animal care facility at Dalhousie University. Mice were ~ 16 months of age when the experiments started (*n* = 17 females, 19 males), to model the age where hypertension is seen clinically [29]. Mice were housed in groups of 2–5 per cage in the Carlton Animal Care Facility at Dalhousie University and maintained on a 12-h light–dark cycle. Mice were fed a Standard Grain-Based Control Rodent Diet (F4059; Bio-Serv, Frenchtown, NJ) and had free access to food and water. All experiments were approved by the Dalhousie University Committee on Laboratory Animals. The care and use of experimental animals followed the Animal Research: Reporting In Vivo Experiments (ARRIVE) guidelines [30].

### Osmotic minipump implantation

Osmotic minipumps (model 2006; Alzet Co) were filled with either saline or AII (Bachem, Cat# 1705) (3 mg/kg/day) and incubated in saline at 37 °C for 24 h before implantation for 6 weeks. This time frame was selected based on protocols used in earlier studies in younger mice [31, 32]. The dosage of AII was chosen based on our pilot studies that determined this dose of AII-induced robust hypertension in mice. Mice were anesthetized with 3% isoflurane (maintained at 2%) and injected with a combination of saline and meloxicam (0.5 mg/kg, subcutaneous) at a 1:1 ratio. Osmotic minipumps were implanted subcutaneously on the animal’s back for 6 weeks. Mice were monitored for humane endpoint (HEP) weight (80% initial weight plus minipump weight) following the implantation procedure. Mice were euthanized with an overdose of sodium pentobarbital (200 mg/kg; IP) if their HEP weight was reached.

### Blood pressure and echocardiography

BP and echocardiography were measured at the baseline (week 0), and then again at the endpoint (week 6) of the study. BP was assessed using the IITC Life Sciences tail-cuff system for mice (Woodland Hills, CA). Mice were initially adapted to the BP procedure for 2 days before experimental data were collected. Briefly, mice were acclimatized to the BP cuff for 10 min and then subjected to 3–5 rounds of five BP measurements. Final BP measurements were carried out in a similar fashion on the third day. Systolic BP and diastolic BP were used to calculate mean arterial pressure. Mean arterial pressure = [(2 × diastolic BP) + systolic BP]/3.

Echocardiography was performed with the Vevo 2100 imaging system (Visual Sonics) and a high-resolution linear transducer (18–38 MHz, MS400). Mice were anesthetized with 3% isoflurane (maintained at 1–1.5%) and their chest hair was removed with a depilatory cream. Body temperature was maintained at 35–36 °C using a heated platform plus heat lamp. Heart rate and respiration were monitored with the Vevo 2100 image system. Short axis M-mode measurements including heart rate, EF, fractional shortening, and corrected left ventricle mass (LV mass) were collected from each mouse. Left ventricle posterior wall (LVPW) thickness, left ventricle anterior wall (LVAW) thickness, and left ventricular internal diameter (LVID) were measured in both systole and diastole. In addition, four-chamber apical Doppler measurements including E wave, A wave, E/A ratio, isovolumic relaxation time (IVRT), and isovolumic contraction time (IVCT) were obtained. Images were analyzed using Vevo LAB software (Visual Sonics).

### Quantitative polymerase chain reaction

At the end of the experiment, mice were euthanized with an overdose of sodium pentobarbital (200 mg/kg; IP) and their hearts were isolated, flash-frozen in liquid nitrogen, and stored at -80°C until use. For qPCR, total RNA was isolated using a commercial Aurum Total RNA Fatty and Fibrous Tissue Kit (Cat # 7326830; Bio-Rad, Mississauga, ON) following the manufacturer’s instructions. Briefly, frozen ventricle samples were homogenized in Trizol with a bead mill homogenizer (Omni Bead Ruptor12, Cole Parmer, Montreal, QC) and total RNA quantity and quality were evaluated with a Nanodrop Lite spectrophotometer (Thermo Fisher, Waltham, MA).

Isolated mRNAs were further converted to cDNA by using Reverse Transcription Supermix for RT-qPCR (Cat # 1708840; Bio-Rad) cDNA synthesis kit. Previously validated and optimized primer pairs for collagen 1 (Col1a1), collagen 3 (Col3a1), fibronectin (Fn1), transforming growth factor beta (Tgfb1), Cd80, Mcp-1, Cd68, and Mrc1 were obtained from Integrated DNA Technologies (Coralville, IA). BLAST and melt curve analyses were performed to ensure primer specificity. The expression of Col1a1, Col3a1, Fn1, Tgfb1, Cd80, Mcp-1, Cd68, and Mrc1 was measured with SsoAdvanced Universal SYBR Green Supermix and CFX Connect Real-Time PCR Detection System (Bio-Rad). The qPCR reaction conditions were as follows: initial polymerase activation and denaturation at 95°C for 30 s, denaturation at 95°C for 15 s, and annealing and extension at 60°C for 40 s. Data were analyzed using CFX Maestro software (Bio-Rad). Data were initially normalized to the reference gene Rplp0, which was stable under all experimental conditions. To permit comparisons between sexes, all samples were normalized against a single control group, in this case the male controls. Primer information can be found in Supplementary Table S1.

### Western blotting

Frozen tissues were homogenized in radioimmunoprecipitation (RIPA) lysis buffer with protease inhibitor cocktails (Santa Crutz Biotechnology, Inc., TX, U.S.A.; # sc24948) using the bead mill homogenizer. Lysates were cleared by centrifuging at 16,000 × g for 20 min at 4°C, and the supernatant was collected. The total protein concentration of the supernatant was measured with a bicinchoninic acid protein assay (Thermo Fisher Scientific Inc. Mississauga, ON #23227). Monoclonal antibodies for collagen 1, collagen 3, fibronectin, and TGF-β were purchased from Santa Cruz Biotechnology (#sc293182, #sc514601, #sc8422, #sc130348). Samples were denatured by boiling with Laemmli sample buffer (#161073; Bio-Rad, Mississauga, ON, CA) containing 355 mM 2-mercaptoethanol at 95°C (5 min). Equal aliquots (20 µg) of total protein were electrophorized on 4–15% or 7% Mini-PROTEAN™ TGX Stain Free™ Protein Gels (Bio-Rad, Mississauga, ON, CA). The fractionated proteins were electrophoretically transferred to 0.45-µM pore-sized nitrocellulose membrane (#1620115, Bio-Rad, Mississauga, ON, CA) at 100 V (1 h) using transfer buffer (48 mM Tris, 39 mM glycine, pH 9.2, 0.03% SDS, and 10% methanol) with an ice pack. Stain-free transferred blots were imaged with a ChemiDoc imaging system (Bio-Rad, Mississauga, ON, CA) to ensure proper transfer. Blots were blocked with 3% fat-free milk (for 1 h at room temperature) and hybridized with collagen 3 (1:900), collagen 1 (1:900), TGF-β1(1:1000), and fibronectin (1:800) antibodies overnight at 4°C. Blots were washed three times for 5 min each in Tris-buffered saline (TBS; 20 mM Tris and 150 mM NaCl) with 0.1% Tween20 (Sigma-Aldrich MO, USA; #P1379). Blots were further hybridized with goat anti-mouse IgG (H + L) antibody (Abcam Inc. MA, USA; #ab97023; 1:10,000) conjugated with horseradish peroxidase at room temperature (1 h). Blots were washed again three times for 5 min each with TBS and 0.1% Tween 20 and then chemiluminescence was developed with the Clarity™ Western ECL Substrate kit (Bio-Rad, Mississauga, ON, CA; #1705060). Images were recorded with the ChemiDoc imaging system (Bio-Rad, Mississauga, ON, CA). Total protein on stain-free blots was used to ensure equal loading, and we also normalized each protein of interest to total protein. To quantify protein, densitometric scanning and analyses of blots were completed with Image Lab Software version™ 6.0 (Bio-Rad Laboratories Inc, 2017). For each Western blot, we ran the gels at the same time, using identical and simultaneous handling for both gels, including using the same buffers and same transfer protocols, and we ran the gels in the same cassette together, for both the separation and transfer. Two samples, control female 1 (F1) and AII female 1 (F1), were run as technical replicates on both gels for each protein to ensure there were no inter-blot variations. The intensities for these two samples were similar and the average for each sample was used in subsequent analyses.

### Statistical analysis

Data obtained from this study were expressed as the mean ± standard error of mean (SEM). We performed outlier tests using a Grubbs test. We removed no more than one outlier per data set for a total of three data points removed in the study. We also tested our data for normality using Shapiro–Wilk, and in a few cases Kolmogorov–Smirnov tests. All datasets were normally distributed except the pre-treatment E wave data for male controls in Fig. 4C. Given that this was the only non-normally distributed data set, it was in the control group, and three-way ANOVA with mixed models is robust to violations of normality [33], we did not modify our analytical approach to this data set. Echocardiography data were analyzed with three-way ANOVA/mixed-effects analysis followed by Fisher’s Least Significant Difference (LSD) post-hoc test for specific comparisons with GraphPad Prism 10.0 (GraphPad Software, Inc.). Molecular data were analyzed with 2-way ANOVA followed by a Fisher’s LSD post hoc test with Sigma Plot 14 (Systat Software, Inc.). Specific tests used for each data set are indicated in the figure legends. Graphs were created with Sigma Plot 14. Values of *p* < 0.05 were considered significant in all analyses.

## Results

### Impact of chronic AII on BP in both male and female mice

**Fig. 1:**
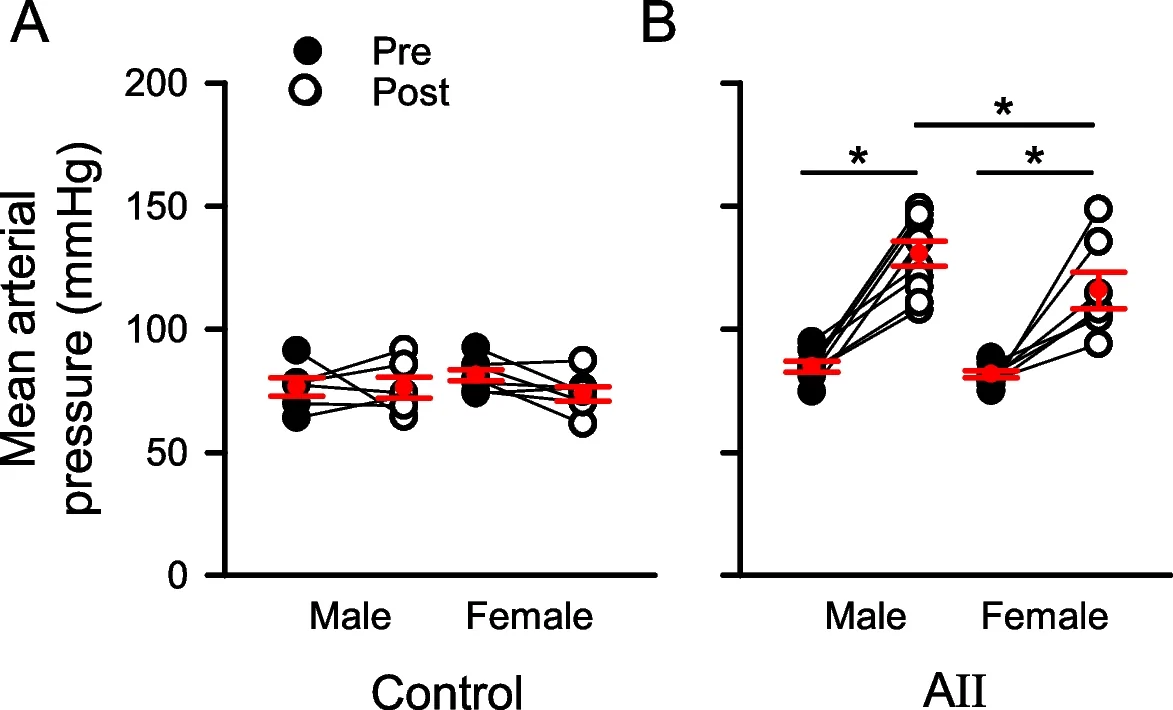
AII infusion led to hypertension in mice of both sexes. **A** Mean arterial pressure was not altered over the course of the study in mice implanted with control minipumps. **B** However, mean arterial pressure increased significantly over time in AII-treated male and female mice compared to baseline measurements. In each case, data illustrate responses before (pre) and after (post) infusion of saline (control) or AII. Sample sizes were 6 control and 10 treated for males, with 8 control and 10 treated females. Data were analyzed with a three-way ANOVA mixed-effects analysis and a Fisher’s LSD post hoc test. Individual data points are illustrated and differences between groups were considered significant if *p* < 0.05, as denoted by the * symbol. Means ± SEM are illustrated in red

We first confirmed the increase in BP caused by AII infusion in older male and female mice. Figure 1A shows that there was no change in mean arterial pressure over the 6 weeks of the study in mice implanted with control osmotic minipumps. By contrast, mean arterial pressure was markedly elevated in both male and female mice after 6 weeks exposure to AII (Fig. 1B). Mean arterial pressure increased by 1.5-fold in treated males and by 1.4-fold in treated females. Similar results were seen when we compared the impact of AII on systolic and diastolic BP (Supplementary Table S2). These findings show that chronic exposure to AII caused a robust increase in BP in aging mice of both sexes.

### Impact of AII on cardiac structure and function assessed with M-mode echocardiography

**Fig. 2:**
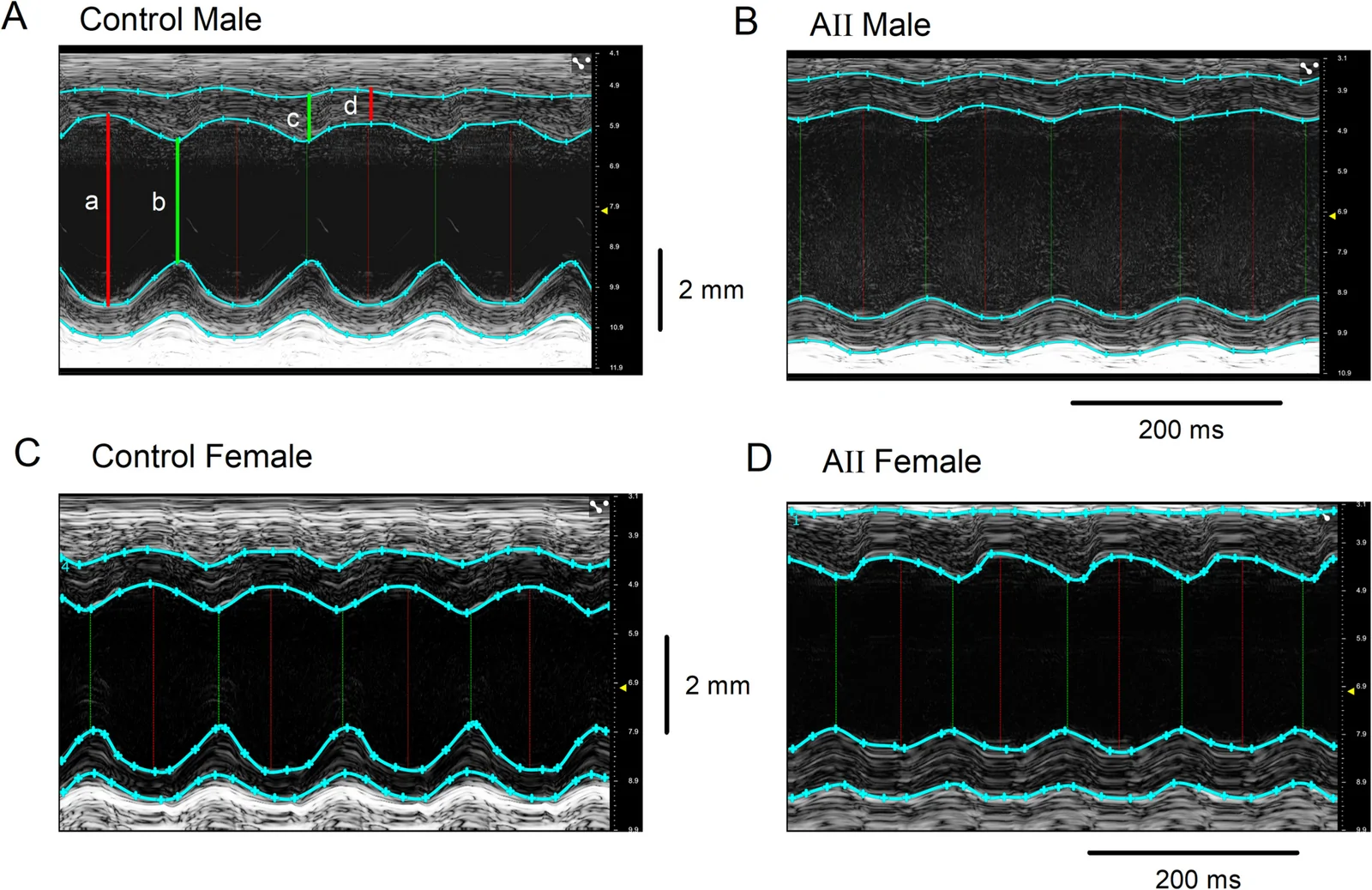
M-mode echocardiography images from aging male and female hearts under control conditions and after exposure to AII. Representative M-mode echocardiography images of hearts from a male mouse mice implanted with a control osmotic minipump (**A**) and with an AII-containing minipump (**B**) at the end of the 6-week study. Corresponding M-mode images recorded from control (**C**) and AII-treated (**D**) female mice. The letters a, b, c, and d represent diastolic diameter, systolic diameter, systolic anterior wall thickness, and diastolic anterior wall thickness, respectively

**Fig. 3:**
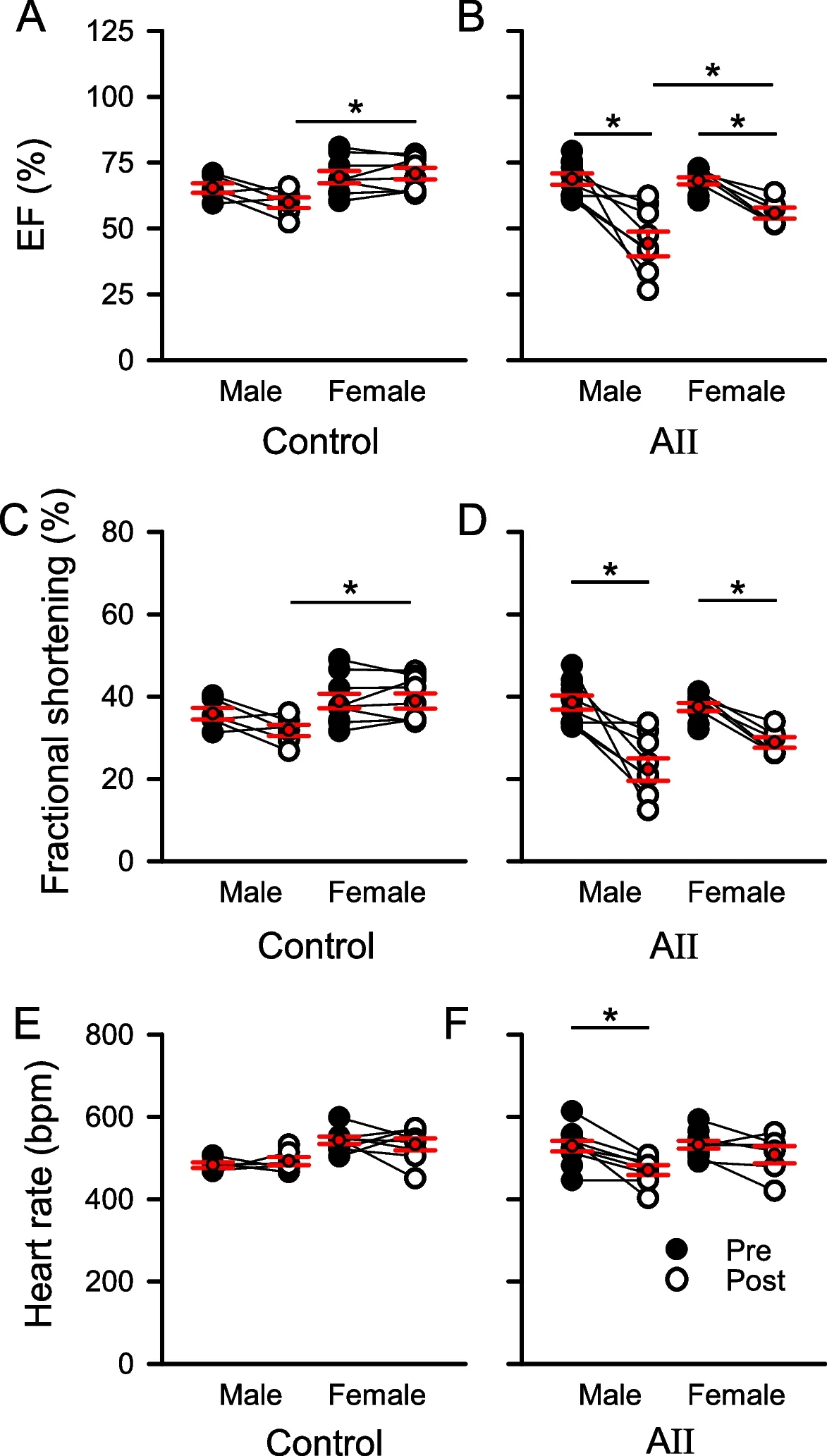
Chronic AII exposure reduced ejection fraction and fractional shortening, especially in males and reduced heart rate in males only. **A** Ejection fraction was not altered by time in the control mice, although it was higher in females than males. **B** AII reduced ejection fraction in both sexes, but this effect was significantly greater in males compared to females. **C** Fractional shortening was greater in females than in males under control conditions. **D** Fractional shortening was reduced in AII-treated hearts, but this effect was more prominent in AII-treated males than in treated females. **E**, **F** Heart rate was largely unaffected in any group, although it did decline in AII-treated males. In each case, data illustrate responses before (pre) and after (post) infusion of saline (control) or AII. Sample sizes were 6 control and 8–11 treated for males, with 8–9 control and 6–10 treated females. Data were analyzed with a three-way ANOVA mixed-effects analysis and a Fisher’s LSD post hoc test. Individual data points are illustrated and differences between groups were considered significant if *p* < 0.05, as denoted by the * symbol. Means ± SEM are illustrated in red

To explore male–female differences in AII-mediated maladaptive remodeling of the aging heart, we used in vivo echocardiography to investigate the impact of AII infusion on ventricular structure and function in older mice of both sexes. Figure 2 shows representative M-mode images recorded from a male mouse implanted with a control minipump (Fig. 2(A)) and a male mouse implanted with an AII-containing minipump (Fig. 2(B)). Corresponding representative examples of M-mode images recorded from females are shown in Fig. 2(C, D). Figure 2 illustrates the anterior (top) and posterior (bottom) walls of the LV, showing how they move as a function of time as the heart beats. Figure 3 shows mean M-mode echocardiography functional data from control and AII-treated mice of both sexes. While EF was not altered by time in the control mice, it was significantly higher in females than in males (Fig. 3A). AII treatment caused a dramatic decrease in ejection fraction in hearts from males (Fig. 3B). By contrast, AII caused only a modest decrease in this parameter in female hearts, and mean EF remained above 50% (Fig. 3B). This sex difference in the impact of AII on EF was statistically significant (Fig. 3B). As seen with EF, fractional shortening was higher in control females when compared to males (Fig. 3C). AII also reduced fractional shortening in both sexes, an effect that was pronounced in the males (Fig. 3D). AII had little effect on heart rate in any group, although it did cause a small decrease in heart rate in the older males (Fig. 3E, F). These results indicate that, while AII promoted systolic dysfunction in both sexes, this effect was predominantly seen in older male mice. Average EF remained well above 50% in AII-treated females.

**Fig. 4:**
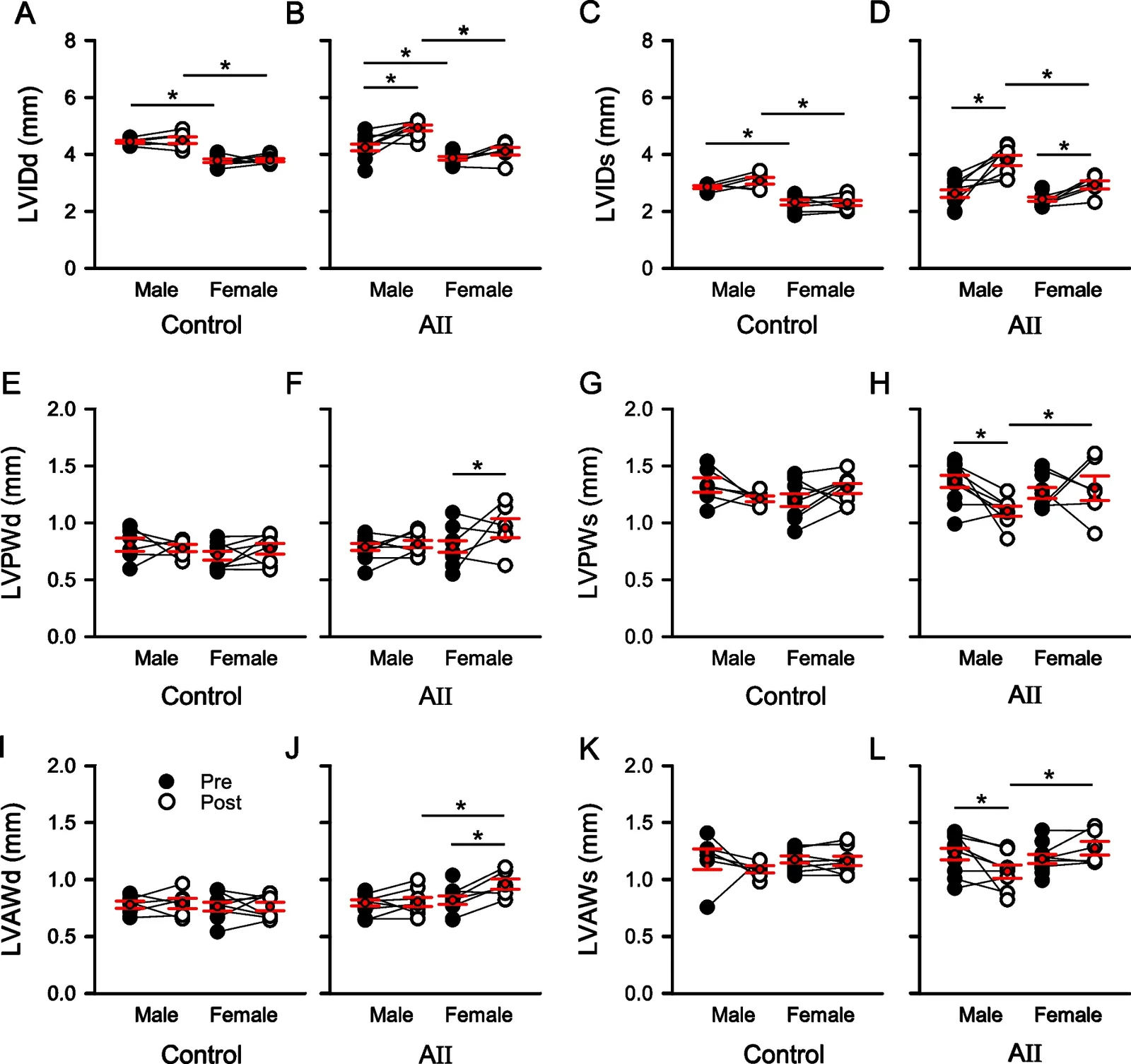
AII infusion increased LV mass in both sexes, but it increased LV diameter in males only and increased LV wall thickness in females only. **A** In control mice, LV internal diameter in diastole (LVIDd) was larger in males than females. **B** AII treatment increased LVIDd in males but not in females.** C** LVID in systole (LVIDs) was larger in control males than in control females. **D** LVIDs increased in AII-infused males and females, but this effect was significantly greater in males than in females. **E** LV posterior wall thickness in diastole (LVPWd) was similar throughout the experiment in control mice. **F** AII treatment increased LVPWd in female but not male mice. **G** LVPW in systole (LVPWs) was similar in all groups in control experiments. **H** AII treatment reduced LVPWs in males but not it in females, such that it was larger in treated females than males. **I**, **J** As with LVPWd, LV anterior wall thickness in diastole (LVAWd) was unaffected in control mice but was markedly increased by AII treatment in female mice only. **K** LVAW in systole (LVAWs) was not affected by time in control. **L** As with LVPWs, AII decreased LVAWs in males but not in females. In each case, data illustrate responses before (pre) and after (post) infusion of saline (control) or AII. Sample sizes were 6 control and 8–11 treated for males, with 8–9 control and 6–10 treated females. Data were analyzed with a three-way ANOVA mixed-effects analysis and a Fisher’s LSD post hoc test. Individual data points are illustrated and differences between groups were considered significant if *p* < 0.05, as denoted by the * symbol. Means ± SEM are illustrated in red

We also examined the influence of chronic AII infusion on structural parameters assessed with M-mode echocardiography. We first assessed the impact of AII on the inner diameter of the LV to determine if AII-treated hearts showed any chamber enlargement. Figure 4A shows that control male hearts had larger internal diameters than control female hearts. Intriguingly, AII treatment increased the LVID in diastole (LVIDd), but this occurred only in males (Fig. 4B). AII had similar effects on LVID in systole (LVIDs), although there was a small increase in this parameter in female hearts (Fig. 4C, D). Next, we assessed parameters that relate to cardiac hypertrophy, starting with LV wall thickness. LVPW in diastole (LVPWd) did not change over time in control mice (Fig. 4E), but AII-infusion increased LVPWd in female mice only (Fig. 4F). Interestingly, LVPW in systole (LVPWs) actually decreased in response to AII in males and values were significantly lower than in treated females (Fig. 4G, H). LVAW in diastole (LVAWd) was maintained in control mice (Fig. 4I) and increased in response to AII only in females (Fig. 4J). Interestingly, LVAW in systole (LVAWs) decreased in AII-treated males and values were significantly lower than in treated females, but there were no effects in controls (Fig. 4K, L). As there is evidence that echocardiography does not accurately measure mass in the aging heart [34], we measured mass directly by weighing the hearts (Table 1). There was an overall effect of sex on body weight where males in both groups were heavier than females. We also found that both raw heart weight and heart weight normalized to tibia length were larger in males than females in control. Infusion of AII increased both unnormalized and normalized heart weight in both sexes (Table 1). Together, these findings indicate that there are clear sex-specific differences in AII-induced structural remodelling, where AII increased ventricular wall thickness primarily in females but reduced wall thickness and led to LV dilation predominantly in males.

**Table 1:** Selected morphometric parameters for mice used in this study

Parameter	Control		AII-treatment	
	Male	Female	Male	Female
Heart weight (g)	0.21 ± 0.01	0.15 ± 0.01^b^	0.25 ± 0.01^a^	0.21 ± 0.02^a,b^
Body weight (g)	39.72 ± 1.71	30.19 ± 1.88^c^	35.01 ± 1.03	29.31 ± 1.05^c^
Tibia length (mm)	18.60 ± 0.11	18.42 ± 0.14	18.42 ± 0.10	18.49 ± 0.09
Heart weight/tibia length (mg/mm)	11.30 ± 0.01	8.34 ± 0.30^b^	13.70 ± 0.01^a^	11.40 ± 0.01^a,b^
Sample size	7–9	8–11	11	10–12

^a^Significantly different from control

^b^Significantly different from male

^c^Overall effect of sex is significant; *p* < 0.05. Values represent the mean ± standard error of mean (SEM)

### Influence of AII on cardiac function as measured with Doppler echocardiography

**Fig. 5:**
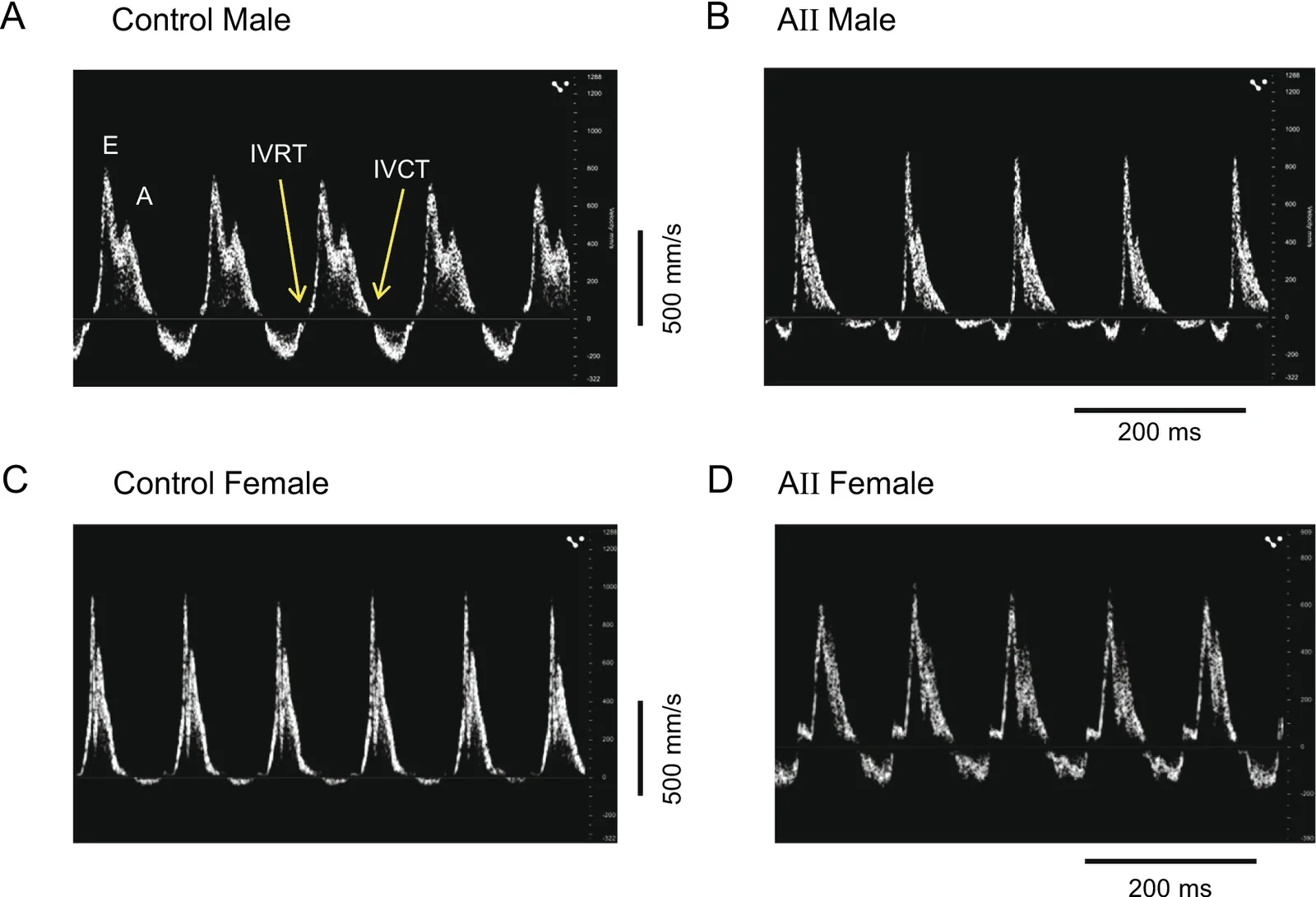
Doppler echocardiography images from aging male and female hearts under control conditions and after exposure to AII. Representative Doppler echocardiography images of in vivo LV function in male mice implanted with a control osmotic minipump (**A**) or an AII-containing minipump (**B**). Corresponding M-mode images recorded from control (**C**) and AII-treated (**D**) female mice. Images were recorded at the end of the 6-week study

**Fig. 6:**
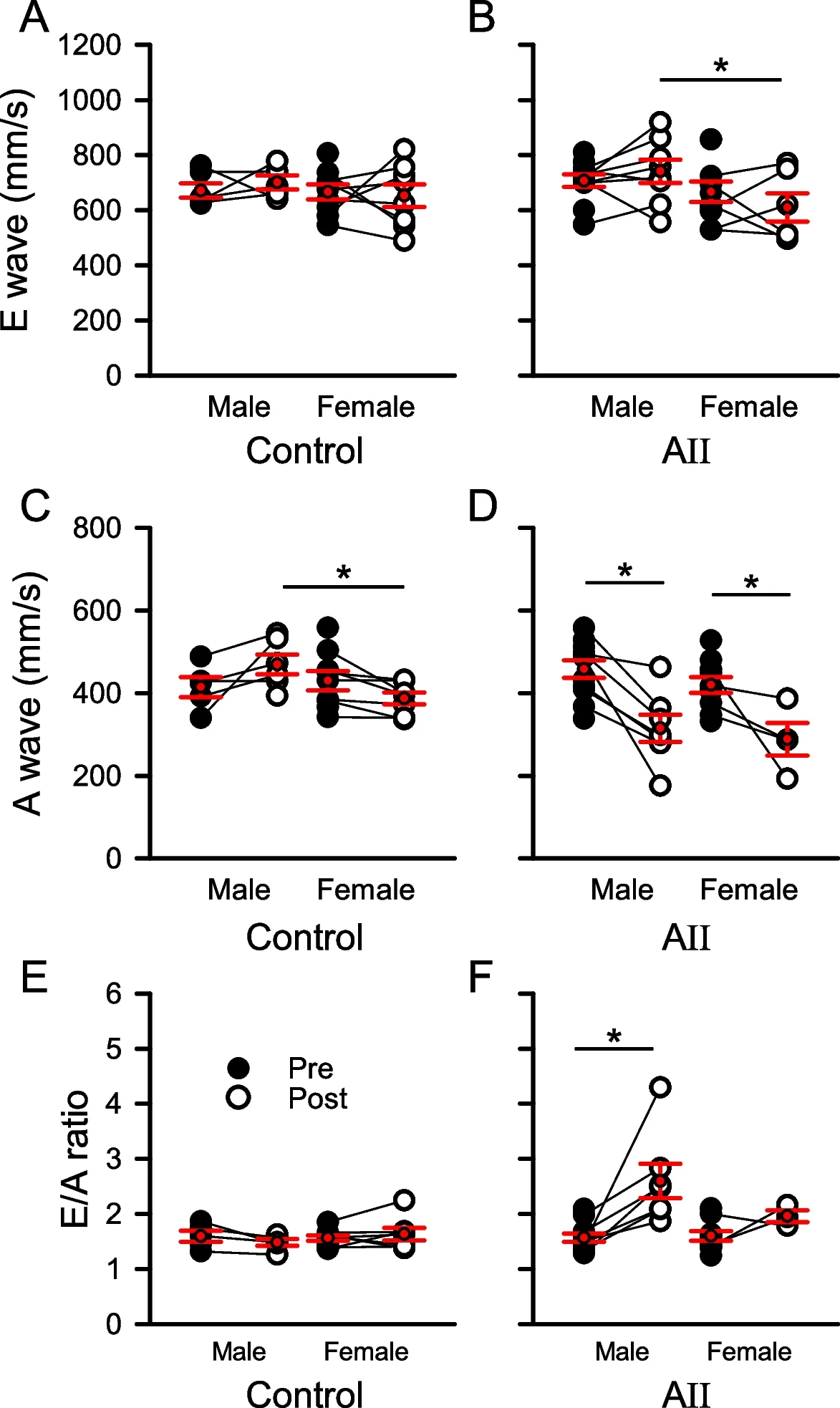
Chronic AII treatment increased E/A ratios in male mice only. **A** In control mice, E waves were similar in both sexes. **B** AII treatment increased the magnitude of E waves in males but not females. **C** In control mice, A waves were slightly smaller in females than in males. **D** AII treatment reduced peak A waves in both sexes. **E** E/A ratios were not affected by time in control mice. **F** AII infusion increased E/A ratios in male mice only. In each case, data illustrate responses before (pre) and after (post) infusion of saline (control) or AII. Sample sizes were 5–6 control and 6–11 treated for males, with 7–9 control and 4–10 treated females. Data were analyzed with a three-way ANOVA mixed-effects analysis and a Fisher’s LSD post hoc test. Individual data points are illustrated and differences between groups were considered significant if *p* < 0.05, as denoted by the * symbol. Means ± SEM are illustrated in red

**Fig. 7:**
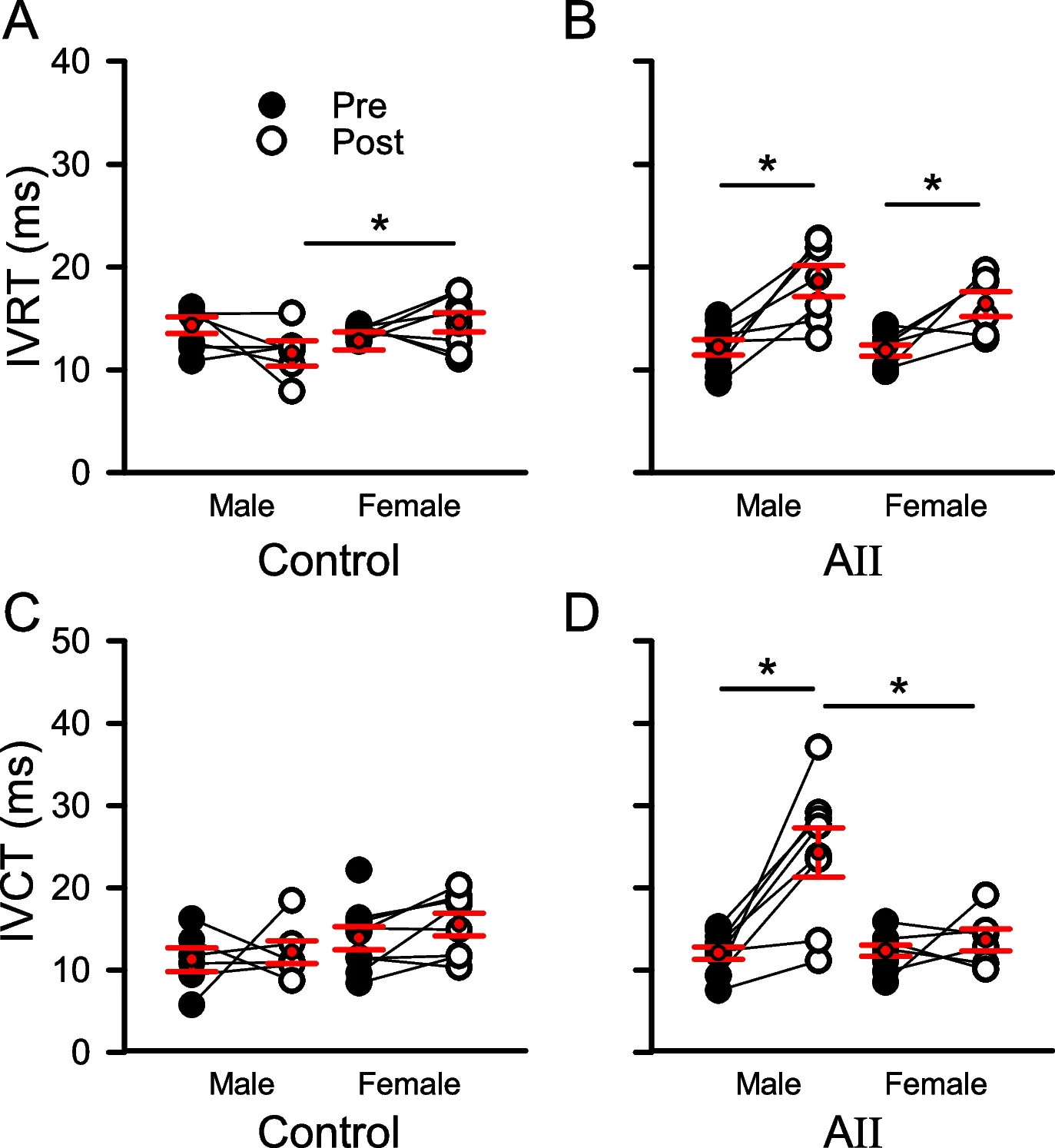
Chronic exposure to AII prolonged IVRT in both sexes, but it disrupted systolic function in male mice only. **A** Isovolumic relaxation time (IVRT) was slightly longer in control females than in control males. **B** AII increased IVRT in both sexes. **C** Isovolumic contraction time (IVCT) was not affected during the experiment in the control group. **D** Treatment with AII increased IVCT in males only. In each case, data illustrate responses before (pre) and after (post) infusion of saline (control) or AII. Sample sizes were 5–6 control and 6–11 treated for males, with 7–9 control and 4–10 treated females. Data were analyzed with a three-way ANOVA mixed-effects analysis and a Fisher’s LSD post hoc test. Individual data points are illustrated and differences between groups were considered significant if *p* < 0.05, as denoted by the * symbol. Means ± SEM are illustrated in red

We next used pulse-wave Doppler echocardiography to investigate potential sex-specific impacts of AII on in vivo cardiac remodeling in hearts from older mice. Representative Doppler images recorded from a control male mouse and an AII-treated male mouse are shown in Fig. 5A and B. Pulse-wave Doppler images recorded from females are shown in Fig. 5C and D. Figure 5 illustrates E (early filling velocity) waves and A (late filling velocity) waves as well as IVRT and IVCT. Mean data are shown in Figs. 6 and 7. We first compared the impact of AII on E and A waves. In control mice, we found that E waves were similar in both sexes (Fig. 6A) and that AII treatment led to larger E waves in males only (Fig. 6B). A waves were slightly smaller in females under control conditions (Fig. 6C) and exposure to AII-reduced A waves in both sexes (Fig. 6D). As higher E/A ratios can indicate diastolic dysfunction [35], we next compared the E/A ratios between groups. We found that E/A ratios were similar across the study in control mice (Fig. 6E) and AII treatment increased E/A ratios in males but not females (Fig. 6F). As prolonged IVRT also is indicative of diastolic dysfunction [35], we next examined the impact of AII on IVRT measured from the Doppler recordings as indicated in Fig. 5. Under control conditions, IVRT was slightly longer in females than in males, but only at the end point, not at baseline (Fig. 7A). However, IVRT was clearly prolonged by AII treatment in both sexes (Fig. 7B). Increases in IVCT are associated with systolic dysfunction [36], so we assessed the impact of AII on IVCT. Results showed that IVCT was unchanged over the course of the study in controls (Fig. 7C). However, IVCT was markedly prolonged by AII in male hearts only and this sex difference was statistically significant (Fig. 7D). Taken together, these findings suggest that chronic infusion of AII promotes diastolic dysfunction in hearts from older mice of both sexes but leads to systolic dysfunction predominantly in older males.

A complete summary of the echocardiography data described above, presented as the mean ± SEM, can be found in Supplementary Table S2. The complete set of *p* values for the mixed effects analysis of all cardiac parameters measured with M-mode echocardiography can be found in Supplementary Table S3. The corresponding mixed effects analysis of the Doppler echocardiography data can be found in Supplementary Table S4.

### Chronic AII elevates mRNA for pro-inflammatory M1 and anti-inflammatory M2 macrophage markers in a sex-specific manner

**Fig. 8:**
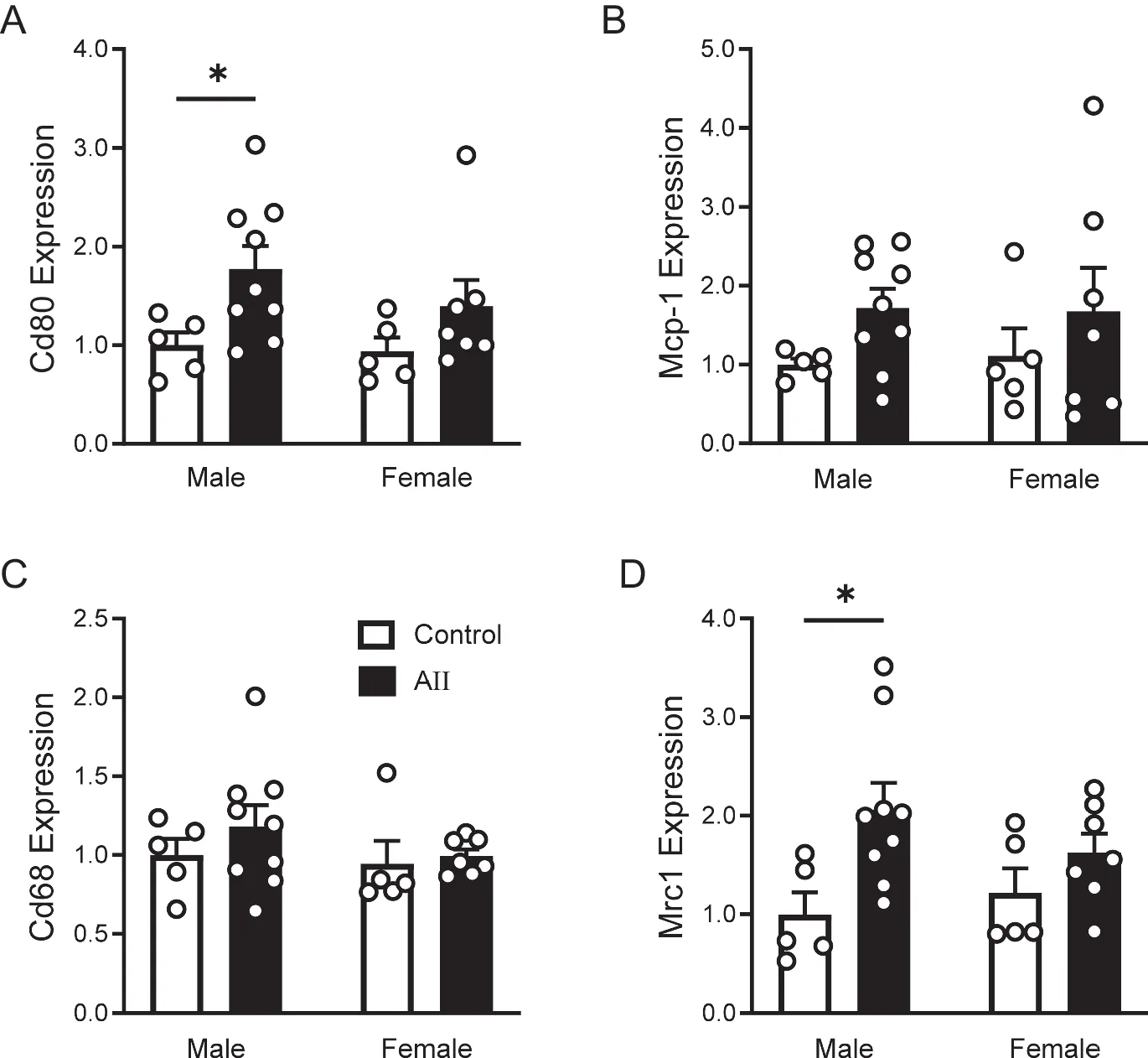
Sex-specific effects of chronic AII infusion on mRNA for M1 and M2 macrophage markers in the ventricle. **A** mRNA levels for the M1 macrophage marker Cd80 were higher in AII-treated males but not females. **B**, **C** mRNA levels for the M1 macrophage markers Mcp1 and Cd68 did not change in any group. **D** AII increased the mRNA abundance for the M2 macrophage marker Mrc1 in males but not in females. Data were first normalized to a reference gene (RPLP0). All samples were then normalized relative to the mean value in male controls. Sample sizes were 5 control and 9 treated males, with 5 control and 7 treated females. Data were analyzed with two-way ANOVA, followed by a Fisher’s LSD post hoc test. Individual data points are illustrated and differences between groups were considered significant if *p* < 0.05, as denoted by the * symbol

As high BP enhances inflammation [4], which plays a role in adverse cardiac remodeling [3], we compared the effects of AII on pro- (M1) and anti-inflammatory (M2) macrophages in ventricles from male and female hearts. Our qPCR results showed that AII infusion led to a 1.8-fold increase in mRNA levels for the M1 macrophage marker Cd80 in male, but not female hearts when compared to controls (Fig. 8A). By contrast, mRNA levels for two other M1 macrophage markers (McpP-1 and Cd68) were similar in control and AII-treated mice regardless of sex (Fig. 8B, C). AII infusion also led to a twofold increase in the abundance of mRNA for the anti-inflammatory M2 marker (Mrc1) in male but not female animals (Fig. 8D). These observations indicate that levels of mRNA for macrophages that mediate both pro- (Cd80) and anti- (Mrc1) inflammatory responses are increased in ventricles from aging male mice chronically exposed to AII. This effect of AII was sex-specific, as these changes were not seen in ventricles from older female mice treated with AII.

### Chronic exposure to AII increases expression of profibrotic markers in a sex-specific fashion

**Fig. 9:**
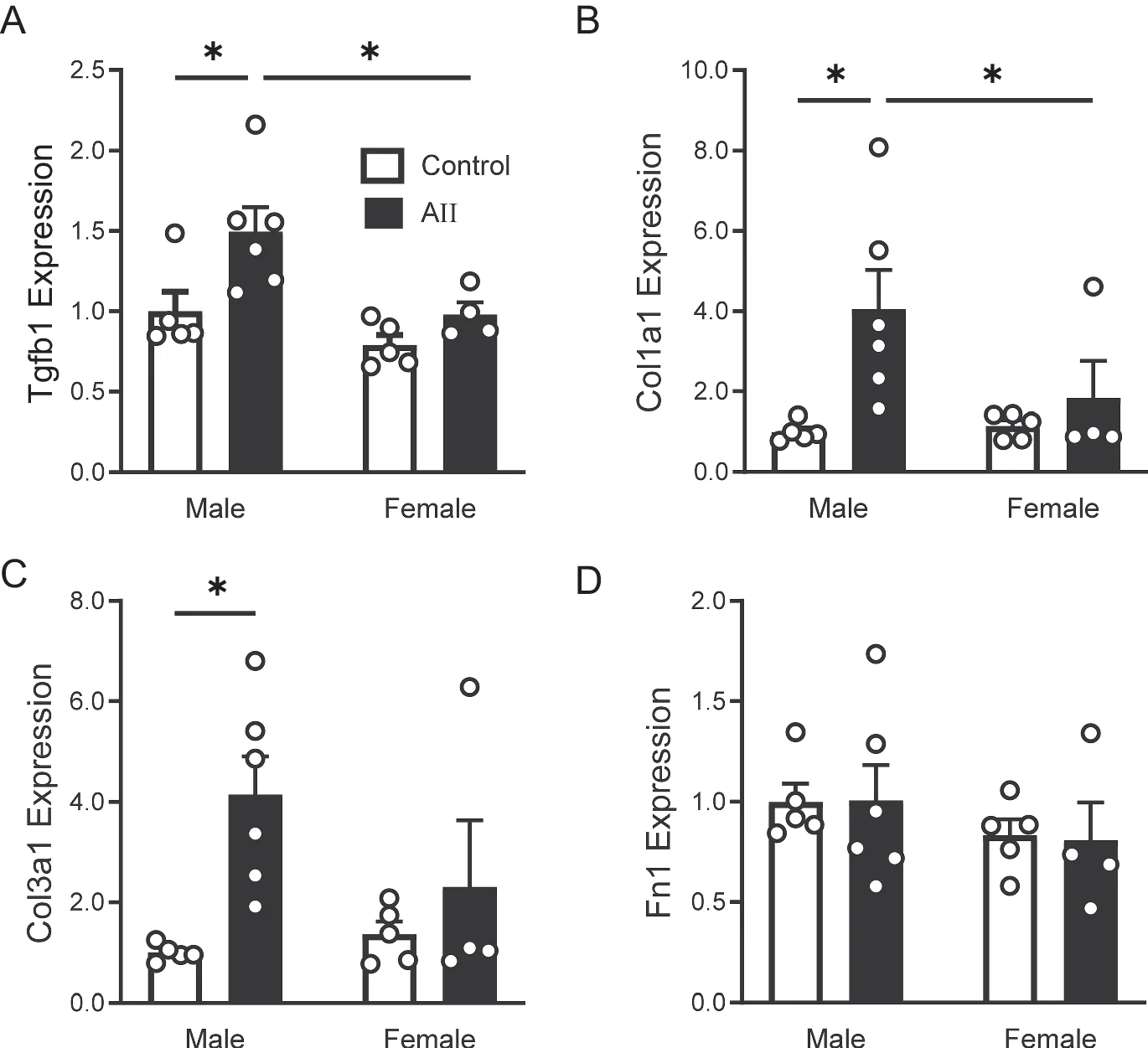
Chronic exposure to AII increased mRNA for the profibrotic markers Col1a1, Col3a1, and Tgfb1, but only in male ventricles. **A** mRNA levels for the profibrotic marker Tgfb1 were increased by AII treatment in males only, and levels were significantly lower in treated females than in treated males. **B**, **C** The levels of mRNA for both Col1a1 and Col3a1 were significantly higher in ventricles from AII-treated males compared to control. Col1a1 levels also were significantly lower in treated females than in treated males. **D** The levels of mRNA for Fn1 were similar in control and AII-treated hearts regardless of the sex of the animal. Data were first normalized to a reference gene (RPLP0). All samples were then normalized relative to the mean value in male controls. Data were normalized to a reference gene (RPLP0) and are illustrated relative to controls. Sample sizes were 5 control and 6 treated males, with 5 control and 4 treated females. Data were analyzed with two-way ANOVA, followed by a Fisher’s LSD post hoc test. Individual data points are illustrated and differences between groups were considered significant if *p* < 0.05, as denoted by the * symbol

The observed changes in macrophages involved in inflammatory responses may activate profibrotic signaling and lead to progressive tissue damage in the heart [37]. Therefore, next we investigated whether the expression of profibrotic mediators (Col1a1, Col3a1, Fn1, Tgfb1) in the ventricles was affected by AII and whether this differed between the sexes. Our qPCR data showed that mRNA levels for Tgfb1, Col1a1, and Col3a1 were significantly increased in AII-treated males when compared control males (Fig. 9A, B, C). By contrast, there was no difference in the levels of mRNA for these three profibrotic mediators in hearts from AII-treated aging females (Fig. 9A, B, C). This difference between treated males and females was statistically significant for Tgfb1 and Col1a1 (Fig. 9A, B). Levels of mRNA for Fn1 were not affected by chronic exposure to AII in either sex (Fig. 9D).

**Fig. 10:**
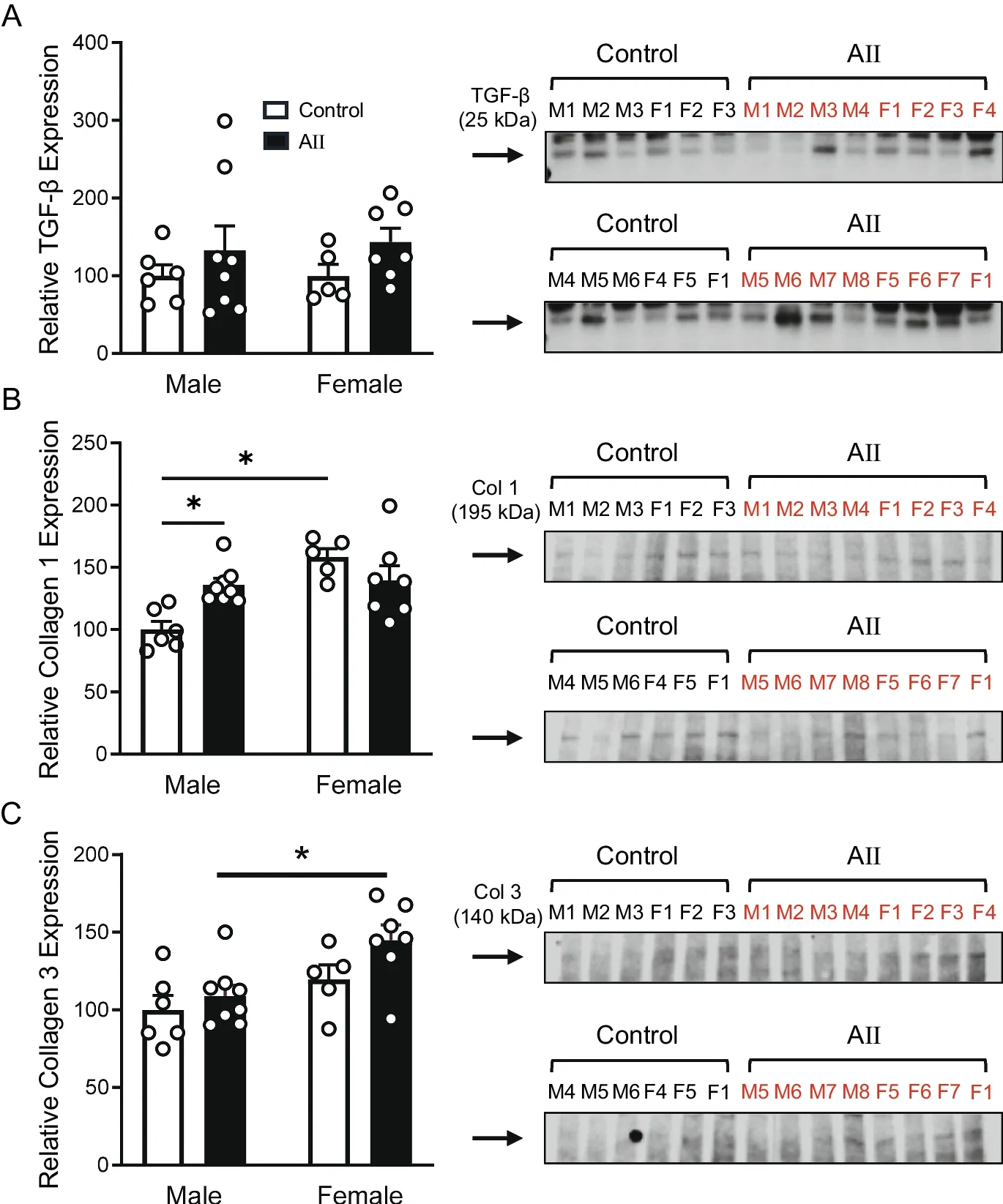
AII increased the expression of collagen 1 protein in male hearts and AII treatment increased collagen 3 in female hearts relative to treated males. **A** The levels of TGF-β protein were not affected by AII treatment, regardless of sex. **B** Collagen 1 protein expression was increased by AII in males. Collagen 1 levels were also higher in female controls when compared to male controls. **C** Collagen 3 protein levels were higher in treated females than in treated males. The corresponding Western blots are shown next to each panel. Data were normalized to total protein and are presented relative to male controls. Sample sizes were 6 control and 8 treated males, with 5 control and 7 treated females. Two samples, control female 1 (F1) and AII female 1 (F1), were run as technical replicates on both gels for each protein. Data were analyzed with 2-way ANOVA, followed by a Fisher’s LSD post hoc test. Individual data points are illustrated and differences between groups were considered significant if *p* < 0.05, as denoted by the * symbol. Samples from AII treated mice are labelled red

In the next series of experiments, we compared protein levels for these profibrotic markers in control and AII-treated hearts to determine if expression differed between the sexes. Data were normalized to total protein (Supplementary Fig. 1) and are presented relative to male control data. Although TGF-β protein expression was slightly higher in both sexes, this effect was not statistically significant (Fig. 10A). However, collagen 1 protein levels in males were significantly higher in AII-treated hearts when compared to controls (Fig. 10B). By contrast, AII had no effect on collagen 1 protein levels in females, but collagen 1 was significantly higher in control females than in control males (Fig. 10B). AII also increased the levels of collagen 3 protein in female ventricles and this difference between AII-treated males and females was statistically significant (Fig. 10C). We were unable to detect fibronectin in any sample with Western blots, but our qPCR results suggest that Fn1 was not affected by AII (Fig. 9D). Taken together, these findings suggest that chronic exposure to AII increases collagen 1 in ventricles from aging males but not females, while AII treatment increases collagen 3 in females when compared to AII treated males. This may contribute to fibrosis and systolic dysfunction in the male heart in the setting of aging.

## Discussion

This study investigated whether cardiac remodeling induced by AII differed between the sexes in older C57BL/6 mice and explored underlying mechanisms. We found that 6 weeks of AII treatment caused a pronounced increase in BP in both sexes. However, AII-induced structural and functional remodeling of the ventricle differed importantly between the sexes. While AII increased signs of LV hypertrophy such as wall thickness predominantly in females, it led to LV dilation and reduced wall thickness primarily in males. Diastolic dysfunction, characterized by elevated E/A ratios and prolonged IVRT, was observed in males while females exhibited an increase in IVRT only. However, male hearts exhibited marked systolic dysfunction associated with reduced EF and a profound prolongation of IVCT. By contrast, EF was preserved above 50% and IVCT was unaffected in female hearts. Our qPCR results showed increases in mRNA for M1 (Cd80) and M2 (Mrc1) macrophages involved in inflammatory responses in AII-treated male but not female ventricles. We also found an increase in mRNA for pro-fibrotic markers (Col1a1, Col3a1, Tgfb1) in male but not in female ventricles. This male-specific increase in collagen 1 also was observed at the protein level, but AII increased collagen 3 in females when compared to AII-treated males. There was also a baseline sex difference in collagen 1 protein levels, where control females had higher levels than control males. Together, these results demonstrate that chronic exposure to AII has sex-specific adverse effects in the ventricles from older mice.

We found that chronic infusion of AII led to signs of diastolic dysfunction in hearts from older male and female mice. This included prolonged IVRT in both sexes and an increase in E/A ratios in males only. These signs are consistent with diastolic dysfunction [35] and indicate that AII slows ventricular relaxation and promotes diastolic dysfunction in the setting of aging. Previous studies report that pressor doses of AII either have no effect on IVRT or they increase IVRT in young (8–12-week-old) male mice [38, 39]. Our results extend these observations to demonstrate that AII markedly increases IVRT in the setting of aging, and that this occurs in both sexes. Unlike the increase in E/A ratios seen in our work, pressor doses of AII actually reduce E/A ratios in young male mice [38–41]. It is important to note that there are grades of diastolic dysfunction, where lower E/A ratios indicate mild diastolic dysfunction and higher ratios indicate profound dysfunction [42]. Thus, previous studies suggest that exposure to AII produces only minimal diastolic dysfunction, at least in young male mice [38–41]. However, the large increase in E/A ratios seen in our work indicates that AII infusion leads to marked diastolic dysfunction in older male mice. As diastolic dysfunction can precede systolic dysfunction [35], early diastolic changes may set the stage for systolic dysfunction in later life.

A critical finding in our study is the observation that chronic exposure to AII produced profound systolic dysfunction in hearts from aging male mice but had much less effect on systolic function in aging females. Indeed, we found that EF was markedly reduced and IVCT was increased by AII treatment in males. By contrast, IVCT was unaffected in females and EF remained above 50%. A reduction in EF and increase in IVCT are associated with poor systolic function [36, 43]. In addition, increases in IVCT predict the occurrence of HFrEF clinically [36, 43]. This suggests that AII treatment promotes severe systolic dysfunction in males but not females, and this may predispose aging male hearts towards HFrEF. In addition to the functional changes we observed, the structural changes seen in male hearts are compatible with a HFrEF phenotype. For example, the LV dilation and thinner ventricular walls seen in AII-treated male hearts is characteristic of eccentric hypertrophy, as typically seen in HFrEF [44]. Our findings are compatible with male–female differences seen clinically, where older men are much more likely to develop HFrEF than older women [27]. 

In contrast to males, we found few signs of systolic dysfunction in older female mice. EF declined only slightly in female mice exposed to AII and remained above 50% at the end of the study. In addition, AII had no impact on IVCT in hearts from older females. This suggests that, despite a robust increase in BP due to AII infusion, systolic function was relatively preserved in older female mice. Females also exhibited diastolic dysfunction, which is compatible with a HFpEF phenotype. The structural changes we observed in older female hearts also are consistent with this idea [44]. For example, there were signs of concentric hypertrophy in AII-treated female hearts, including thicker LV walls with no increase in LV chamber volumes, as seen in HFpEF [44]. These observations concur with clinical findings, where HFpEF is much more common in older women than in age-matched men [28].

Chronic RAS activation promotes inflammation that contributes to adverse cardiac remodeling [3, 4, 10]. Therefore, we investigated whether markers for macrophages involved in pro- (M1) and anti-inflammatory (M2) responses were affected by AII and whether this differed between the sexes. Interestingly, we found an increase in the M1 macrophage marker Cd80 in ventricles from males but not females. As M1 macrophages release pro-inflammatory cytokines and promote tissue damage [45], an increase in AII-mediated inflammation in male hearts may contribute to the adverse functional and structural remodeling observed in our study, although additional studies will be required to test this directly. Interestingly, the M2 macrophage marker Mrc1 also was elevated in ventricles from male mice. As M2 macrophages are involved in anti-inflammatory responses and repair of damaged tissues [45], it is possible that compensatory responses to reduce inflammation are activated in the aging male heart. Although M1 and M2 macrophage markers were elevated in AII-treated ventricles from older males, this was not observed in age-matched females. The reasons for this sex difference are unclear. While both sexes exhibit age-related immune dysfunction, older males exhibit more profound maladaptive changes in immune function than age-matched females [46]. In addition, the degree of systemic chronic inflammation (known as inflammaging) is higher in older males than in older females [46]. In theory, this may serve to augment male responses to immunoinflammatory challenges like exposure to AII. However, many other factors including hormonal status, the environment and overall health also can promote immune dysfunction and inflammaging, and these may also influence male–female differences in responses to AII [46, 47].

AII-induced inflammation can convert cardiac fibroblasts to myofibroblasts, which increases synthesis of extracellular matrix proteins (e.g., collagens) leading to cardiac fibrosis [6–9, 48]. We therefore investigated markers of fibrosis including Col1a1, Col3a1, Tgfb1, and Fn1 to determine if they were affected by AII. Levels of mRNA for Fn1, an extracellular matrix component that is activated before collagen deposition [49], were not affected by AII in either sex. However, mRNA for Tgfb1, a growth factor that converts fibroblasts to myofibroblasts, and mRNA for the primary cardiac Col1a1 and Col3a1 [48] was increased in hearts from male but not female AII-treated mice. This suggests that markers of cardiac fibrosis are increased by AII treatment in male but not female hearts.

The corresponding collagen 1 protein levels also were increased by AII treatment in male hearts when but not in female hearts. In terms of impact on the aging male heart, the AII-induced increase in collagen 1 would be expected to increase ventricular fibrosis and promote systolic dysfunction [48, 50, 51] as we observed in this study. We also found that AII treatment increased collagen 3 protein in females compared to collagen 3 protein levels in males. As collagen 3 is deposited earlier than collagen 1 during cardiac fibrosis [52], it is possible that AII would increase collagen 1 over a longer time frame in females. On the other hand, overexpression of collagen 3 prevents systolic dysfunction, LV dilatation, and ventricular fibrosis in a rodent model of ischemic heart disease [53]. Thus, this may be a beneficial change in the female heart that helps mitigate adverse effects of AII. We also found that the levels of collagen 1 protein were higher in control females than in control males. Interestingly, previous work has shown that, while young female hearts have less collagen 1 than males, collagen 1 increases with age due to an increase in fibroblast proliferation in female but not male hearts [54]. This may account for the higher levels of collagen 1 protein in female control mice when compared to male controls. However, it is evident from our findings that collagen 1 does not further increase in response to AII in older female hearts. We also saw an increase in mRNA for Tgfb1 in treated males, with no corresponding increase in protein. Perhaps if we had waited longer, we would have observed an increase in TGF-β protein.

There are limitations to the work presented here. The results of our qPCR and Western blot studies clearly indicate that AII induced markers of fibrosis in aged male but not female mice; however, it would be of interest to quantify the extent of fibrosis with Picrosirius red or Trichrome staining in future work. We used 16-month-old mice [55] to approximate the age of onset of hypertension (e.g., mean age of onset of 46 years) seen clinically [29]. In future work, it would be interesting to include younger mice to determine if the observed sex difference emerges at earlier ages. It would also be of interest to determine whether adjusting the AII dose to a more extended treatment period would exacerbate these male–female differences in cardiac remodeling. AII had little effect on heart rate, although it caused a small decrease in older males. It is important to note that heart rate was measured in anesthetized mice undergoing echocardiography and isoflurane can affect heart rate in C57Bl/6 mice [56]. Direct electrocardiogram recordings in unanesthetized mice would be of interest. We also found that AII caused a slightly larger increase in BP in males (1.5-fold) than in females (1.4-fold). Although it is possible that some sex-specific effects of AII could relate to this small difference in BP, this seems unlikely given the small magnitude of the difference in BP compared to the large sex differences in cardiac remodeling reported here. Finally, some echocardiography measurements were variable, especially in females. For example, the IVRT is longer in control females than males at the end of the study but not at baseline. In addition, AII increased LVPWd in females only. While this observation should be interpreted cautiously, the increase in LVAWs is convincing and supports the idea that LV wall thickness increases in response to chronic AII in females. A larger sample size for these parameters would have been helpful.

Our study has evaluated the impact of AII on cardiac remodeling in relevant preclinical models (e.g., older mice of both sexes). As previous preclinical studies of AII remodeling have used young, mostly male animals, our findings help fill an important knowledge gap. It seems clear that both age and sex are critical in setting the stage for the expression of late life illnesses like cardiovascular diseases. Preclinical studies of chronic conditions, like cardiovascular diseases, should include older animals of both sexes to better mimic the individuals who are most likely to develop these diseases as they age. Our findings have potential clinical significance. We showed that chronic exposure to AII at this age resulted in adverse cardiac remodeling featuring both structural (hypertrophy, dilation, inflammation, and fibrosis) and functional (systolic and diastolic dysfunction) impairment that differed in many respects between the sexes. For example, aged male mice have increased E/A ratios and prolonged IVRT suggestive of diastolic dysfunction in response to after AII infusion, while older females exhibit only prolonged IVRT. In addition, AII induces profibrotic responses in males only. This suggest that the molecular mechanism involved in diastolic dysfunction may differ between the sexes. Future studies should explore whether there are female-specific impairments in calcium handling and/or myofilament proteins, both of which are implicated in diastolic dysfunction [57]. A greater understanding of these underlying mechanisms may help identify new sex-specific therapeutic strategies for HF treatment in older people. At present, treatment of HFrEF with sacubitril–valsartan (neprilysin inhibitor/AII receptor blocker) is recommended for both sexes, but it is not yet clear that this treatment is effective in HFpEF, regardless of sex [58]. As collagen levels were higher in males, therapeutic strategies targeting cardiac fibrosis may be beneficial in older men. Additional studies to explore this concept would be of interest.

In summary, older male mice exposed to chronic AII infusion exhibit profound systolic dysfunction with LV dilation and thinning of the ventricular walls, changes that are not seen in older females. This appears to be mediated, at least in part, by enhanced profibrotic signaling in older male hearts. In contrast, AII-treated females exhibit increased LV wall thickness, with EF preserved at values well above 50%. These findings are important as they provide mechanistic insights into the higher prevalence of HFrEF in men and increased prevalence of HFpEF in women [27]. These observations may help explain sex differences in HF in later life. 

## Supplementary Information

Below is the link to the electronic supplementary material.

Supplementary file1 (PDF 1220 KB)

Supplementary file2 (DOCX 58 KB)

## Data Availability

Data will be made available upon reasonable request.
