# Supplementary material for: Angiotensin II induces sex-specific ventricular remodeling in aging C57Bl/6 mice
Source: GeroScience. 2025 Nov 7;48(4):5721–40. doi: 10.1007/s11357-025-01924-y (PMC13575036; doi:10.1007/s11357-025-01924-y)

Supplemental Figure 1

Total protein for TGF-β

A

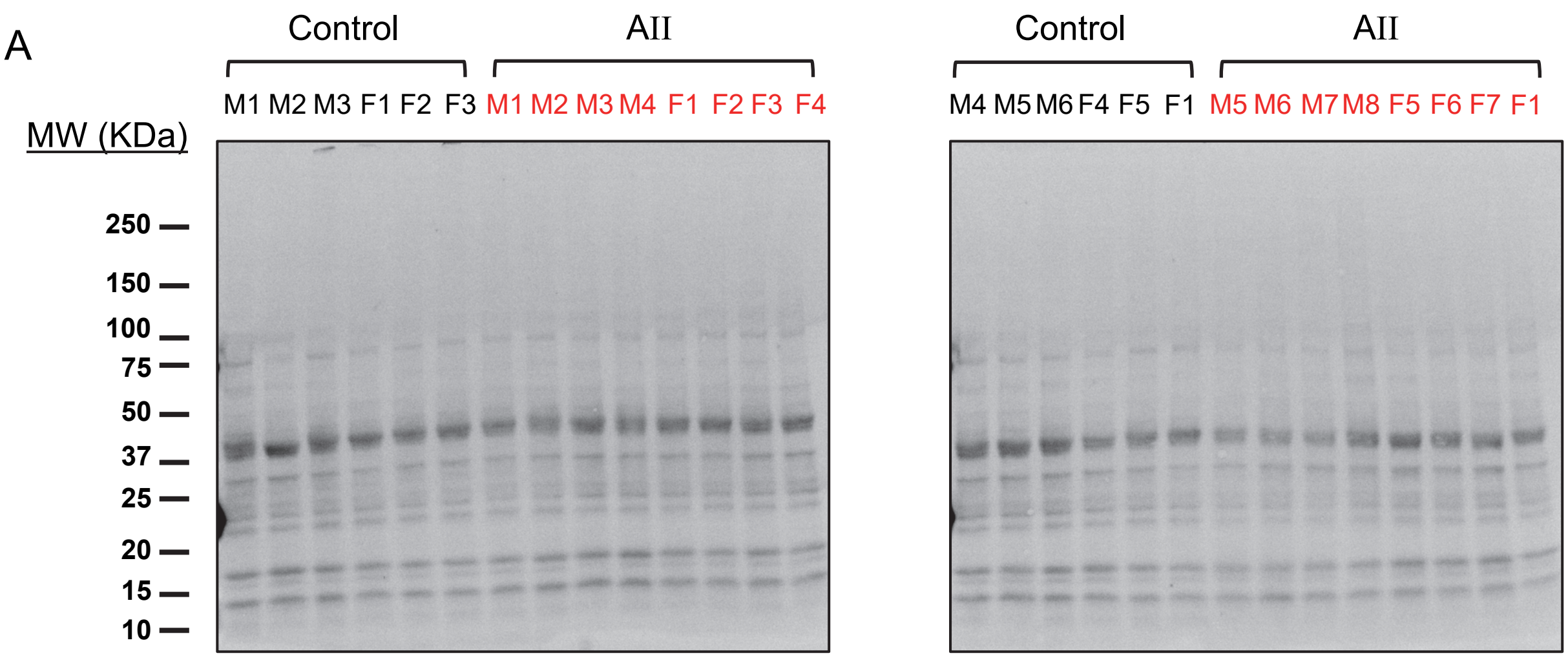

Total protein for Collagen 1

B

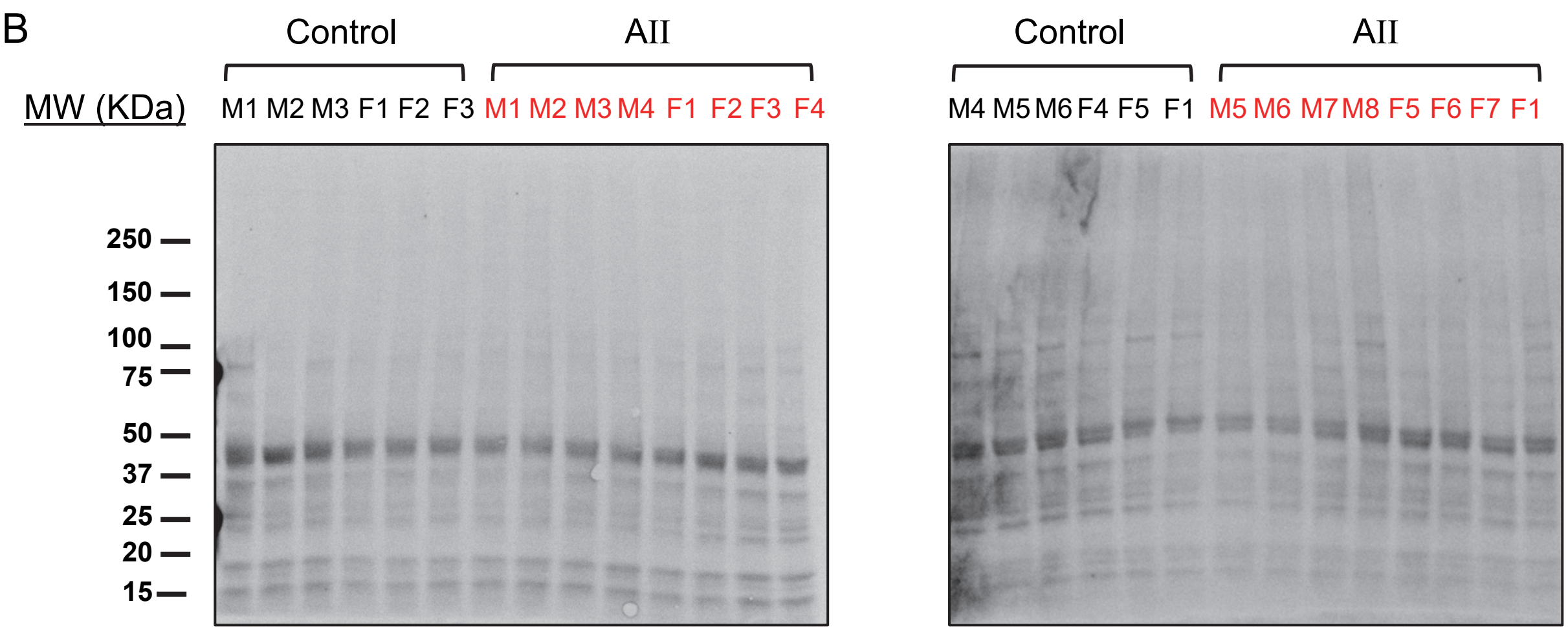

Total protein for Collagen 3

C

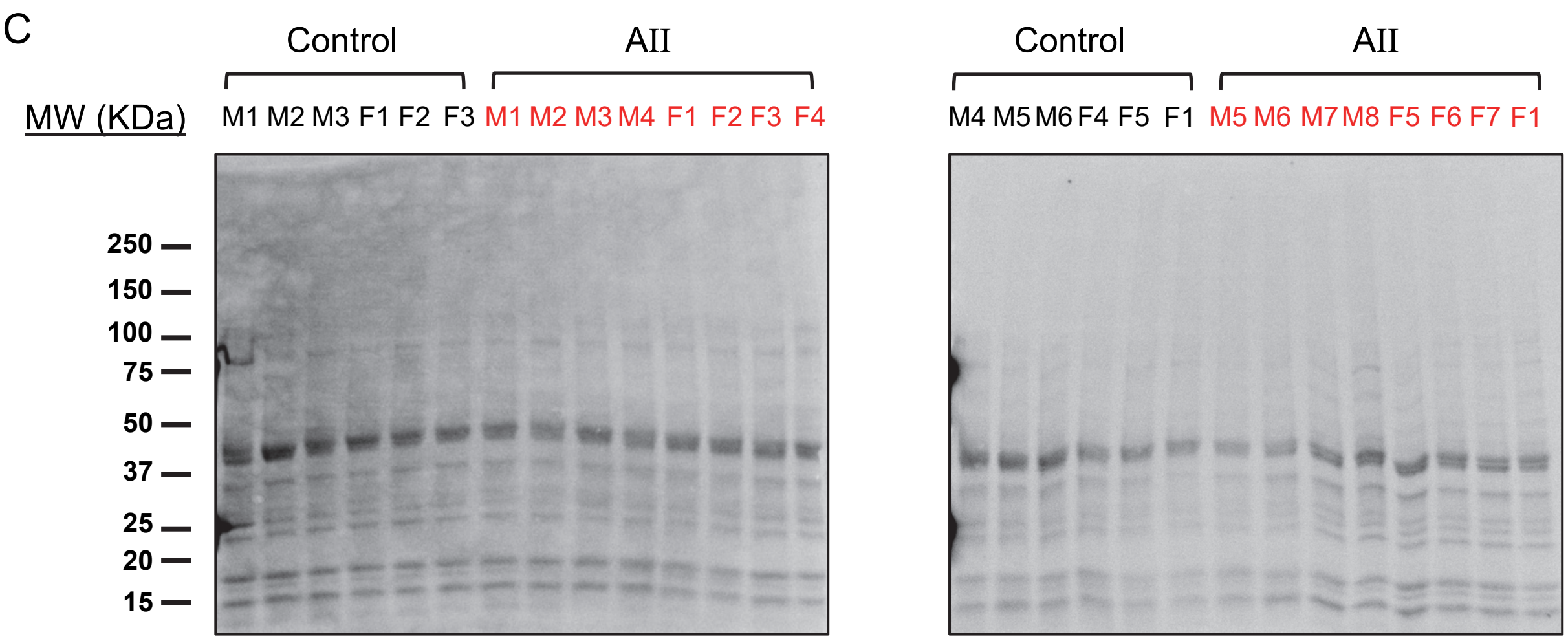

Supplement: Supplementary file 1 [file 11357_2025_1924_MOESM1_ESM.pdf]
