# Supplementary material for: Angiotensin II induces sex-specific ventricular remodeling in aging C57Bl/6 mice
Source: GeroScience. 2025 Nov 7;48(4):5721–40. doi: 10.1007/s11357-025-01924-y (PMC13575036; doi:10.1007/s11357-025-01924-y)
Supplement: Supplementary file 2 [file 11357_2025_1924_MOESM2_ESM.docx]

Sept 5, 2025

**SUPPLEMENTARY INFORMATION**

**ANGIOTENSIN II INDUCES SEX-SPECIFIC VENTRICULAR REMODELING IN AGING C57Bl/6 MICE**

Ninh Khuong^1*^, Manish Mishra^1*^, Shubham Banga^1^, and Susan E. Howlett^1,3^

^*^Both authors equally contributed to this work.

^1^Department of Pharmacology and ^3^ Department of Medicine (Geriatric Medicine), Dalhousie

**Corresponding author:**

Susan Howlett, PhD

Dalhousie University

Department of Pharmacology

PO Box 15000, Halifax, NS, Canada B3H 4R2

Phone: +19024943552

Email: [susan.howlett@dal.ca](mailto:susan.howlett@dal.ca)

**SUPPLEMENTARY FIGURE LEGENDS**

**Supplementary Figure S1: Total protein for the Western blots presented in Figure 10.** Total protein used to normalize TGF-β protein **(A)**, collagen 1 **(B)** and collagen 3 **(C)**. Sample sizes were 6 control and 8 treated males, with 5 control and 7 treated females. Two samples, control female 1 (F1) and AII female 1 (F1), were run as technical replicates on both gels for each protein. Samples from AII treated mice are labelled red.

**SUPPLEMENTARY TABLES**

**Supplementary Table S1:** Gene and primer sequences used in the current study

| **Gene** | **Primer sequence** |
| --- | --- |
| **Col1a1** | Forward 5'-CGC AAA GAG TCT ACA TGT CTA GG-3' |
|  | Reverse 5'-CAT TGT GTA TGC AGC TGA CTT C-3' |
| **Col3a1** | Forward 5'-TTC TTC TCA CCC TTC TTC ATCC-3' |
|  | Reverse 5'-TCT CTA GAC TCA TAG GAC TGA CC-3' |
| **Fn1** | Forward 5'-GAG CTA TCC ATT TCA CCT TCA GA -3' |
|  | Reverse 5'-TTG TTC GTA GAC ACT GGA GAC-3' |
| **Tgfb1** | Forward 5'-GCG GAC TAC TAT GCT AAA GAG G-3' |
|  | Reverse 5'-CCG AAT GTC TGA CGT ATT GAA GA-3' |
| **Cd80** | Forward 5'-TTG TGC TGC TGA TTC GTC TT-3' |
|  | Reverse 5'-TTG CCA GTA GAT TCG GTC TTC-3' |
| **Mcp1** | Forward 5'-CAT CCA CGT GTT GGC TCA -3' |
|  | Reverse 5'-AAC TAC AGC TTC TTT GGG ACA-3' |
| **Cd68** | Forward 5'-CAC CTG TCT CTC TCA TTT CCT T-3' |
|  | Reverse 5'-CCA TGA ATG TCC ACT GTG CT-3' |
| **Mrc1** | Forward 5'-TAT CTC TGT CAT CCC TGT CTCT-3' |
|  | Reverse 5'-CAA GTT GCC GTC TGA ACT GA-3' |
| **Rplp0** | Forward 5'-TTA TAA CCC TGA AGT GCT CGA C-3' |
|  | Reverse 5'-CGC TTG TAC CCA TTG ATG ATG-3' |

**Abbreviations:** Col1a1, Collagen 1; Col3a1, Collagen 3; Fn1, Fibronectin; Tgfb1, transforming growth factor beta; Cd80, Cluster of differentiation 80; Mcp1, Monocyte chemoattractant protein-1, Cd68, Cluster of differentiation 68; Mrc1, Mannose receptor C-type 1; Rplp0, Ribosomal Protein Lateral Stalk Subunit P0.-0

**Supplementary Table S2:** Mean values for echocardiography and blood pressure measurements

| **Parameters, units** | **Male control** | | **Male Ang II** | | **Female Control** | | **Female Ang II** | | **Effect of Ang II** | |
| --- | --- | --- | --- | --- | --- | --- | --- | --- | --- | --- |
|  | **Pre** | **Post** | **Pre** | **Post** | **Pre** | **Post** | **Pre** | **Post** | **♂** | **♀** |
| **SBP** | 95.7 ± 3.9 | 98 ± 3.6 | 101.6 ± 2.6 | 161 ± 6.1**^abc^** | 97.6 ± 3.2 | 95.2 ± 5.0 | 100.9 ± 2.9 | 144 ± 6.9**^abc^** | ↑↑ | ↑↑ |
| **DBP** | 67 ± 4.0 | 65.5 ± 4.8 | 76.6 ± 2.4 | 116 ± 5**^ab^** | 73.3 ± 2.1 | 66 ± 0.7 | 72.3 ± 1.5 | 105.8 ± 8**^ab^** | ↑↑ | ↑↑ |
| **MAP (mmHg)** | 76.6 ± 3.8 | 76.3 ± 4.2 | 84.9 ± 2.2 | 130.7 ± 5.1**^abc^** | 81.4 ± 2.2 | 73.9 ± 2.9 | 81.8 ± 1.4 | 116 ± 7.3**^abc^** | ↑↑ | ↑↑ |
| **HR (bpm)** | 482 ± 6.8 | 492.5 ± 10.2 | 528.9 ± 13.0 | 470.3 ± 12.0**^ab^** | 542.9 ±9.0 | 533 ± 14.5 | 532.2 ± 9.7 | 508 ± 20.8 | ↓ | ↔ |
| **EF (%)** | 65.4 ± 1.8 | 59.9 ± 2.0**^c^** | 68.9 ± 1.9 | 46.1 ± 4.5**^abc^** | 69.5 ± 2.4 | 70.9 ± 2.2**^c^** | 68.2 ± 1.3 | 55.9 ± 2.0**^abc^** | ↓↓ | ↓ |
| **FS (%)** | 35.9 ± 1.4 | 31.8 ± 1.4**^c^** | 38.6 ± 1.5 | 23.5 ± 2.6**^ab^** | 38.9 ±2.0 | 39.9 ± 1.8**^c^** | 37.5 ± 1.0 | 28.9 ± 1.3**^ab^** | ↓↓ | ↓ |
| **LVAWd (mm)** | 0.78 ± 0.03 | 0.79 ± 0.05 | 0.80 ± 0.03 | 0.81 ± 0.04**^ac^** | 0.76 ± 0.04 | 0.77 ± 0.04 | 0.82 ± 0.04 | 0.96 ± 0.05**^abc^** | ↔ | ↑↑ |
| **LVPWd (mm)** | 0.81 ± 0.06 | 0.78 ± 0.03 | 0.79 ± 0.03 | 0.81 ± 0.03 | 0.71 ± 0.04 | 0.77 ± 0.05 | 0.79 ± 0.05 | 0.96 ± 0.08**^ab^** | ↔ | ↑↑ |
| **LVAWs (mm)** | 1.18 ± 0.09 | 1.09 ± 0.03 | 1.23 ± 0.05 | 1.07±0.06**^abc^** | 1.18 ± 0.03 | 1.17 ± 0.04 | 1.18 ± 0.4 | 1.28 ± 0.06**^bc^** | ↓ | ↑ |
| **LVPWs (mm)** | 1.33 ± 0.06 | 1.21 ± 0.03 | 1.37 ± 0.05 | 1.10 ± 0.04**^abc^** | 1.20 ± 0.06 | 1.30 ± 0.05 | 1.30 ± 0.05 | 1.31 ± 0.01**^ac^** | ↓ | ↑ |
| **LVIDd (mm)** | 4.44 ± 0.05**^c^** | 4.50 ± 0.12**^c^** | 4.24 ± 0.12**^ac^** | 4.93 ± 0.10**^abc^** | 3.80 ± 0.06**^c^** | 3.80 ± 0.05**^c^** | 3.9 ± 0.06**^ac^** | 4.11 ± 0.14**^abc^** | ↑↑ | ↔ |
| **LVIDs (mm)** | 2.84 ± 0.05**^c^** | 3.0 ± 0.12**^c^** | 2.62 ± 0.13 | 3.80 ± 0.18**^abc^** | 2.30 ± 0.09c | 2.30 ± 0.09**^c^** | 2.40 ± 0.08 | 2.90 ± 0.14**^abc^** | ↑↑ | ↑ |
| **E** | 672 ± 25.3 | 701.7 ± 25.7 | 707.2 ± 22.6 | 741.3 ± 42.2**^c^** | 666.3 ± 27.2 | 653.1 ± 41 | 667.1 ± 37.3 | 610.2 ± 51.1^c^ | ↑ | ↓ |
| **A** | 415 ± 24.4 | 469.7 ± 24.2**^c^** | 458.4 ± 21.0 | 315.5 ± 33.2**^ab^** | 430.5 ± 23.3 | 387.3 ± 14.7**^c^** | 420.1 ± 19.1 | 288.6 ± 39.6**^ab^** | ↓↓ | ↓↓ |
| **E/A** | 1.6 ± 0.1 | 1.5 ± 0.06 | 1.6 ± 0.08 | 2.6 ± 0.3**^ab^** | 1.6 ± 0.05 | 1.6 ± 0.11 | 1.6 ± 0.09 | 1.6 ± 0.85 | ↑ | ↔ |
| **IVRT** | 13.8 ± 0.9 | 11.7 ± 1.0**^c^** | 12.1 ± 0.7 | 19.0 ± 1.4**^ab^** | 13.7 ± 0.2 | 14.6 ± 0.9**^c^** | 11.9 ± 0.5 | 16.4 ± 1.2**^a^** | ↑↑ | ↑ |
| **IVCT** | 11.3 ± 1.5 | 12.2± 1.4 | 12.1 ± 0.7 | 24.3 ± 3.0**^abc^** | 13.9 ± 1.4 | 15.6 ± 1.4 | 12.4 ± 0.7 | 13.7 ± 1.3**^bc^** | ↑↑ | ↔ |

**Abbreviations:** SBP, systolic blood pressure; DBP, diastolic blood pressure; MAP, mean arterial pressure; EF, ejection fraction; FS, fractional shortening; LVM_(Corrected),_ corrected left ventricle mass; HR, heart rate; bpm, beats per minute; LVAWd, diastolic left ventricular anterior wall; LVPWd, diastolic left ventricular posterior wall; LVAWs, systolic left ventricular anterior wall; LVPWs, systolic left ventricular posterior wall; LVIDs, left ventricular internal diameter end systole; LVIDd, left ventricular internal diameter end diastole; IVRT PW, isovolumic relaxation time pulse wave; IVCT PW, isovolumic contraction time pulse wave; E, E wave; A, A wave; E/A, early filling velocity/ late filling velocity; ↓↓ (major decline); ↓ (minor decline); ↑↑ (major increase); ↑ (minor increase); ≈ (slight increase/decrease or similar); a, significant effect of treatment; b, significant effect of time (pre, post); c, significant effect of sex. p< 0.05.

**Supplementary Table S3: S**ignificance of blood pressure and M-mode echocardiography measures analyzed by mixed effect analysis

| Parameters, unit | Time  (Pre/Post) | Sex | Control  vs  AII | Time x  Sex | Time  x  AII | Sex  x  AII | Time  x  Sex  x  AII |
| --- | --- | --- | --- | --- | --- | --- | --- |
| LVAWs, mm | 0.207 | 0.170 | 0.425 | **0.022** | 0.828 | 0.634 | 0.222 |
| LVAWd, mm | 0.056 | 0.301 | **0.024** | 0.181 | 0.093 | 0.086 | 0.119 |
| LVPWs, mm | 0.153 | 0.721 | 0.960 | **0.002** | 0.225 | 0.401 | 0.622 |
| LVPWd, mm | 0.114 | 0.766 | 0.050 | 0.102 | 0.240 | 0.075 | 0.727 |
| LVIDs, mm | **<0.0001** | **<0.0001** | **0.0008** | **0.011** | **<0.0001** | 0.446 | 0.240 |
| LVIDd, mm | **0.0006** | **<0.0001** | **0.0436** | 0.097 | **0.003** | 0.503 | 0.155 |
| HR, bpm | **0.0143** | **0.0016** | 0.715 | 0.583 | **0.019** | 0.174 | 0.118 |
| EF, % | **<0.0001** | **0.003** | **0.001** | **0.020** | **<0.0001** | 0.395 | 0.630 |
| FS, % | **<0.0001** | **0.011** | **0.002** | **0.027** | **0.0002** | 0.196 | 0.817 |
| SBP | **<0.0001** | 0.1797 | **<0.0001** | 0.1251 | **<0.0001** | 0.2174 | 0.3876 |
| DBP | **<0.0001** | 0.5175 | **<0.0001** | 0.3246 | **<0.0001** | 0.0739 | 0.9857 |
| MAP | **<0.0001** | 0.1776 | **<0.0001** | 0.1042 | **<0.0001** | 0.0822 | 0.6945 |

**Abbreviations:** LVAWs, systolic left ventricular anterior wall; LVAWd, diastolic left ventricular anterior wall; LVPWs, systolic left ventricular posterior wall; LVPWd, diastolic left ventricular posterior wall; LVIDs, left ventricular internal diameter end systole; LVIDd, left ventricular internal diameter end diastole; LV_(Mass Corrected),_ corrected left ventricle mass; HR, heart rate; bpm, beats per minute; EF, ejection fraction; FS, fractional shortening; SBP, systolic blood pressure; DBP, diastolic blood pressure; MAP, mean arterial pressure; p< 0.05 is considered as value of significance in all measurements.

**Supplementary Table S4: S**ignificance of echocardiography Doppler measures analyzed by mixed effect analysis

| Parameters, unit | Time  (Pre/Post) | Sex | Control  vs  AII | Time x  Sex | Time  x  AII | Sex  x  AII | Time  x  Sex  x  AII |
| --- | --- | --- | --- | --- | --- | --- | --- |
| IVRT PW, ms | **0.0001** | 0.731 | **0.018** | 0.590 | **<0.0001** | 0.078 | **0.025** |
| IVCT PW, ms | **0.0009** | 0.344 | **0.044** | **0.027** | **0.017** | **0.0009** | **0.011** |
| E, mm/s | 0.946 | 0.067 | 0.705 | 0.211 | 0.761 | 0.312 | 0.639 |
| A, mm/s | **0.0009** | 0.150 | **0.012** | 0.214 | **0.0002** | 0.987 | 0.079 |
| E/A ratio | **0.004** | 0.259 | **0.0017** | 0.252 | **0.0025** | 0.097 | **0.049** |

**Abbreviations:** IVRT PW, isovolumic relaxation time pulse wave; IVCT PW, isovolumic contraction time pulse wave; E, E wave; A, A wave; E/A, early filling velocity/ late filling velocity. p< 0.05 is considered as value of significance in all measurements.
